# Diverse community of rhizobia-diatom symbioses fixes nitrogen in the South Pacific gyre

**DOI:** 10.1093/ismeco/ycaf207

**Published:** 2025-11-12

**Authors:** Mertcan Esti, Miriam Philippi, Julia Duerschlag, Timothy G Ferdelman, Jennifer Tolman, Julie LaRoche, Clara Martínez-Pérez, Gaute Lavik, Bernhard Tschitschko, Hon Lun Wong, Alexandra Kraberg, Sten Littmann, Abiel T Kidane, Wiebke Mohr, Marcel M M Kuypers

**Affiliations:** Department of Biogeochemistry, Max Planck Institute for Marine Microbiology, 28359 Bremen, Germany; Department of Biogeochemistry, Max Planck Institute for Marine Microbiology, 28359 Bremen, Germany; Alfred Wegener Institute - Helmholtz-Centre for Polar and Marine Research, 27570 Bremerhaven, Germany; Department of Biogeochemistry, Max Planck Institute for Marine Microbiology, 28359 Bremen, Germany; Leibniz Institute, DSMZ-German Collection of Microorganisms and Cell Cultures GmbH, 38124 Braunschweig, Germany; Department of Biogeochemistry, Max Planck Institute for Marine Microbiology, 28359 Bremen, Germany; Department of Biology, Dalhousie University, Halifax, B3H 4R2, NS, Canada; Department of Biology, Dalhousie University, Halifax, B3H 4R2, NS, Canada; Department of Biogeochemistry, Max Planck Institute for Marine Microbiology, 28359 Bremen, Germany; Limnological Research Station, Department of Hydrology, University of Bayreuth, 95447 Bayreuth, Germany; Department of Biogeochemistry, Max Planck Institute for Marine Microbiology, 28359 Bremen, Germany; Department of Biogeochemistry, Max Planck Institute for Marine Microbiology, 28359 Bremen, Germany; Department of Microbiology, University of Innsbruck, 6020 Innsbruck, Austria; Department of Biogeochemistry, Max Planck Institute for Marine Microbiology, 28359 Bremen, Germany; Alfred Wegener Institute - Helmholtz-Centre for Polar and Marine Research, 27570 Bremerhaven, Germany; Department of Biogeochemistry, Max Planck Institute for Marine Microbiology, 28359 Bremen, Germany; Department of Biogeochemistry, Max Planck Institute for Marine Microbiology, 28359 Bremen, Germany; Department of Biogeochemistry, Max Planck Institute for Marine Microbiology, 28359 Bremen, Germany; Department of Biogeochemistry, Max Planck Institute for Marine Microbiology, 28359 Bremen, Germany

**Keywords:** nitrogen fixation, Tectiglobus, rhizobia, diatom symbioses, non-cyanobacterial diazotrophs (NCD), *nifH*, nanoSIMS, South Pacific gyre, marine nitrogen cycle

## Abstract

Nitrogen fixation is crucial for sustaining productivity in most of the open ocean. Cyanobacteria are the most prominent N_2_-fixers, but based on the *nifH* gene, a marker gene of the enzyme that fixes N_2_ into ammonia, non-cyanobacterial N_2_-fixers often predominate the N_2_-fixing community. Yet, the vast majority of them remain poorly characterized. In the oligotrophic South Pacific gyre, we found that most *nifH* gene sequences belonged to non-cyanobacterial N_2_-fixers that dominated the waters with measurable N_2_ fixation rates. Approximately two thirds of the non-cyanobacterial sequences affiliated with the group “Marine 1” which also contains the recently identified diatom symbiont *Ca.* Tectiglobus diatomicola, a heterotrophic bacterium belonging to the order Rhizobiales, and its closest relative, *Ca*. *Tectiglobus profundi*. Using fluorescence *in situ* hybridization and electron microscopy, we found that *Ca*. Tectiglobus-diatom symbioses were present throughout the gyre. These diatom symbioses were also present in samples devoid of *nifH* from *Ca*. *T. diatomicola* and *Ca*. *T. profundi* indicating that other members of the “Marine 1” group are also diatom symbionts. At least two morphologically distinct diatoms harbored *Ca*. Tectiglobus symbionts, revealing a so far unknown diversity in hosts for these rhizobial N_2_-fixers. Single-cell activity measurements showed that *Ca*. Tectiglobus-diatom symbioses actively fixed nitrogen and could account for up to 40% of the N_2_ fixation in the South Pacific gyre. Given the size of the largest oceanic biome and the abundance of *Ca*. Tectiglobus-related *nifH* genes in other ocean regions, these heterotrophic N_2_-fixers likely play a major role in marine nitrogen cycling.

## Introduction

In vast areas of the ocean, primary production is limited by the availability of nitrogen (N) [1–3]. Dissolved dinitrogen (N_2_) gas is abundant, but is biologically available only to diverse but rare N_2_-fixing bacteria and archaea [4]. Until now, marine N_2_ fixation has been attributed mainly to cyanobacteria, such as the filamentous *Trichodesmium*, filamentous *Richelia* living symbiotically with diatoms (diatom-diazotroph-association, DDA), and unicellular cyanobacterial (UCYN) groups, namely UCYN-A, UCYN-B and UCYN-C [5–10]. However, in many ocean regions, cyanobacterial N_2_-fixers cannot fully explain the measured bulk N_2_ fixation rate [9, 11]. In fact, in large areas of the ocean, N_2_ fixation has been reported in the absence or near-absence of known cyanobacterial N_2_-fixers, indicating the activity of non-cyanobacterial N_2_-fixers [12–18]. Molecular analyses of the *nifH* gene, a marker gene for the N_2_-fixing nitrogenase enzyme, indicate a wide diversity and presence of non-cyanobacterial N_2_-fixers in the ocean [13, 19–24]. One of the most abundant and widespread *nifH* phylotypes, gamma-A [22, 25], was recently identified as a member of the alphaproteobacterial order Rhizobiales [26]. This rhizobial N_2_-fixer, *Ca*. Tectiglobus diatomicola, lives symbiotically inside a pennate diatom, *Haslea* sp., with which it exchanges carbon and nitrogen compounds. Such metabolic exchange is also apparent in the UCYN-A-haptophyte symbiosis [9, 10, 27, 28], in which the UCYN-A symbiont was recently postulated to be an N_2_-fixing organelle (or nitroplast [29]). Like UCYN-A-haptophyte symbioses, *Ca*. *T. diatomicola*-diatom symbioses are abundant and contribute substantially to N_2_ fixation in the tropical North Atlantic [9, 26]. However, there is so far no direct evidence that *Ca*. *T. diatomicola* or its close relative *Ca*. *Tectiglobus profundi* also form N_2_-fixing symbioses with diatoms in other ocean regions. As such, it remains unclear how relevant these symbioses are in global marine N_2_ fixation, even though gene sequences related to these rhizobial N_2_-fixers have been detected worldwide [26]. Here, we investigated the occurrence and diversity of *Ca*. Tectiglobus spp.-diatom symbioses in the world’s largest surface water biome, the South Pacific Gyre, where substantial N_2_ fixation occurs despite cyanobacterial N_2_-fixers being rare to non-detectable [11–14, 30].

## Materials and methods

### Sampling and hydrography

**Figure 1:**
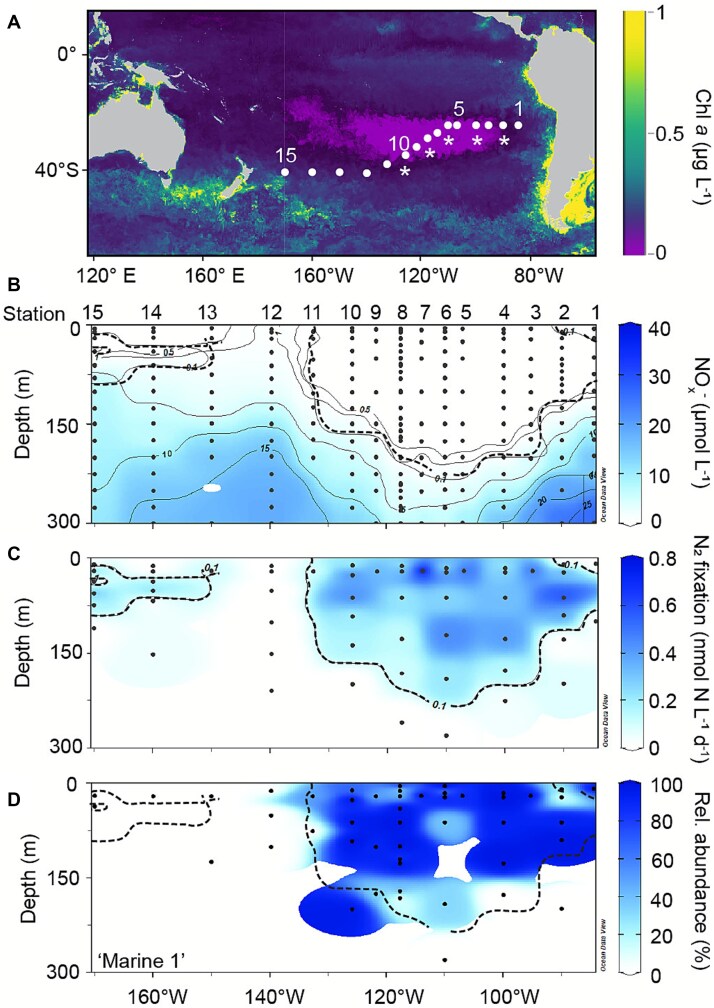
Sampling locations (stations 1–15; circles) throughout the gyre region as shown by the low chlorophyll *a* (chl *a*) concentration in surface waters (monthly composite MODIS image for January 2016; [34]) (A), and depth profiles of nitrate/nitrite (NO_x_^−^) concentration (B), N_2_ fixation rates (average of triplicates) (C) and the relative abundance of *nifH* reads of group “Marine 1” (D) in the South Pacific gyre. Asterisks in (A) indicate stations where *Ca.* Tectiglobus-diatom symbioses were found using fluorescence in situ hybridization in plankton net and/or depth-specific samples. The thick dashed lines in (B), (C), and (D) indicate the isoline of a 0.1 nmol N L^−1^ d^−1^ rate of N_2_ fixation. Data/figures in panels A and B were modified from the original publication in Duerschlag et al. [31] under the Creative Commons License (http://creativecommons. org/licenses/by/4.0/).

Sampling was performed during the R/V Sonne “UltraPac” cruise (SO245) to the South Pacific from Antofagasta, Chile to Wellington, New Zealand (17 December 2015–28 January 2016). At 15 stations, consecutive CTD casts were used to collect samples for nutrient and chlorophyll *a* (chl *a*) concentration, primary production and N_2_ fixation rates and nucleic acids (Fig. 1A). A detailed sampling scheme and primary productivity data can also be found in Duerschlag *et al.* [31], and hydrographic and nutrient data has been deposited with the Pangaea Earth and Environmental Science Data publisher under the DOIs: http://dx.doi.org/10.1594/PANGAEA.899228 and http://dx.doi.org/10.1594/PANGAEA.890394 [32, 33]. In addition to sampling from the CTD casts, we also collected samples from size-fractionated (20–100 μm, 100–180 μm and > 180 μm) plankton net hauls deployed to depths of ~250–300 m at 8 of the 15 stations for later microscopy. Aliquots of the concentrated plankton were briefly stored at 4°C and subsequently filtered onto polycarbonate filters (47 mm diameter, 10 μm pore size; Isopore). Just prior to finishing the filtration, the vacuum was broken and 5 ml of a 2% paraformaldehyde solution were added to the last ~5 ml of plankton sample in the filtration tower, and the sample was left for 1 h at room temperature. After 1 h, the remaining liquid was filtered, and the filter was air-dried and frozen at −20°C until further processing.

### N_2_ fixation rate measurements

N_2_ fixation rates were measured at six depths in the upper water column (surface waters to below the deep chl *a* maximum, DCM) at stations 2, 4, 6, 8, 10, 12, 14, and 15, in surface waters and at ~100 m depth at station 1, and in surface waters at stations 3, 5, 7, 9, 11, and 13. Stable isotope incubations were carried out in triplicate for ~24 h using ^15^N_2_ gas (Cambridge Isotope Laboratories; lot no. I-19168A) added using the bubble removal technique [35, 36] simultaneously with primary production rates that are reported in Duerschlag *et al.* [31]. Bottles were incubated in on-deck incubators, cooled with a continuous flow of surface seawater, and light in the incubators was adjusted to three light levels with Lee filter foils. At the end of the incubation time, subsamples were taken for elemental and isotopic analyses of the biomass as well as microscopy and single-cell analyses. Fixation rates were calculated based on the incorporation of ^15^N into biomass. A more detailed description can be found in the Suppl. Methods.

### Nucleic acid sampling, nucleic acid extraction, and *nifH* amplicon sequencing

Two liters of water were filtered onto polyvinylidene fluoride (PVDF) membrane filters (0.22 μm pore size, 47 mm diameter; Durapore), frozen immediately at −80°C and stored at −80°C. DNA was extracted using the Qiagen AllPrep DNA/RNA Mini Kit with the modifications according to Langlois *et al.* [37]. Partial *nifH* genes were co-sequenced with partial 16S rRNA genes after PCR amplification, and sequences were separated based on primer sequences (16S rRNA primers V4-V5 versus *nifH* primers). The amplicon sequence variants (ASVs) of the 16S rRNA gene sequences were reported in Duerschlag *et al.* [31] and can be found at the NCBI Sequence Read Archive under Bioproject number PRJNA670604.

Prior to *nifH* amplicon sequencing, the extracted DNA samples were screened for the presence of *nifH* genes, and samples with a visible PCR product in a gel electrophoresis were included for *nifH* amplicon library preparation and sequencing. Tag sequencing of the *nifH* DNA amplicons was carried out using 2 × 300 bp paired-end v3 chemistry at the Integrated Microbiome Resource (IMR), Dalhousie University. The raw Illumina paired-end reads of *nifH* were processed with the QIIME pipeline [38] using the Microbiome Helper workflow of the IMR [39]. Briefly, paired-end reads were separated from co-sequenced 16S rRNA gene sequences using amplicon-specific primer sequences. Primers were trimmed using Cutadapt [40], then reads were stitched together using PEAR [41] and denoised into ASVs using Deblur [42] with a trim length of 325 bp and a *nifH* reference set [43]. Sequencing bleed-through was minimized by removing singletons and ASVs with a relative abundance of <0.1% mean sample depth [39]. Further details can be found in the Suppl. Methods.

### Phylogenetic analyses

For *nifH* phylogenetic analyses, *nifH* reference sequences were collected. These included the single best hit for each *nifH* ASV in a blastn search [44] against the NCBI Nucleotide collection (nt; accessed in April 2025), 20 *nifH* amplicon sequences from plankton-associated N_2_-fixers [45], 37 *nifH* sequences from heterotrophic bacterial diazotrophs (HBDs) [24], 26 *nifH* sequences representing different “Gamma” clusters [13, 22, 46], and the single best blastn hit for each *nifH* ASV against a custom full-length *nifH* blast database. This custom full-length *nifH* blast database was based on the full-length *nifH* sequences (over 885 bp) by Heller *et al.* [47] (last updated in 2022) with the addition of two *Ca*. Tectiglobus *nifH*, namely *Ca*. *T. diatomicola* (GCA_039583345) and *Ca*. *T. profundi* (GCA_039793665).

We constructed both nucleotide-based and amino acid-based phylogenetic trees. The amino acid-based tree was used to provide a robust, conservative approach to broader phylogeny of recovered *nifH* amplicon sequences while the nucleotide-based tree was used to study clades within the group “Marine 1” as amino acid-based phylogeny can disguise clades, as can be observed for UCYN-A clades [48]. Trees were visualized and pruned (where applicable) in iTol [49]. Assignment of ASVs to the canonical *nifH* clusters was done using the CART annotation tool [50]. The taxonomy of reference sequences is given only for those *nifH* sequences of known origin (using GTDB (Genome Taxonomy Database) taxonomy) while reference sequences of unknown taxonomic origin were designated as “unknown”. The *nifH* sequence data can be found under Bioproject number PRJEB92104 at the European Nucleotide Archive (ENA) and ASVs can be found in Suppl. Data File 2. Tree files for both the nucleotide-based and amino acid-based trees can be found as Suppl. Data Files 3 and 4. Further details on the phylogenetic analyses and cluster analyses can be found in the Suppl. Methods.

### Quantitative PCR

Three previously published qPCR assays were used to confirm the presence/absence of ASVs belonging to the clades A/B (‘GammaA [37]), 3 (“Gamma3” [13]) and ETSP2 (γETSP2 [46]). In addition, representative synthetic ASV sequences were used to assess the clade specificity of the qPCR assays. For further details, please see Suppl. Methods.

### Recovery of the full-length 16S rRNA gene sequence of *Ca.* Tectiglobus profundi

The metagenome-assembled genome (MAG) of the closest relative of *Ca*. *T. diatomicola*, i.e. *Ca*. *T. profundi* (GCA_039793665) only contained a partial (1108 bp) 16S rRNA gene sequence [26]. In order to assess the specificity of all probes used during fluorescence *in situ* hybridization (see below), we iteratively extended its genome with the aim of recovering the full-length 16S rRNA gene sequence (Suppl. Methods). The refined, full-length 16S rRNA gene sequence can be found in Suppl. Data File 6.

### Fluorescence *in situ* hybridization, microscopy, and single-cell activities using nanoSIMS

Subsamples from the end of the incubation experiments as well as plankton net samples were used to visualized *Ca*. Tectiglobus spp. via fluorescence *in situ* hybridization (FISH) in a double CARD-FISH approach according to Tschitschko *et al.* [26] and adhering to standard procedures [51] as described in the Suppl. Methods. Scanning electron microscopy (FEI Quanta 250 FEG ESEM; Thermo Fisher Scientific) was used to subsequently determine host morphology.

From incubation samples, diatoms with symbionts (FISH-positive cells) were counted from 1–2 filter pieces of ~5 mm diameter each (cut from the 25 mm diameter filters), which equated to 19–31 ml of incubated seawater. A few of these diatoms were further analyzed for their single-cell activity using nanoscale secondary ion mass spectrometry (nanoSIMS). For the plankton net samples, diatoms were only visualized but not quantified due to the uneven distribution of collected plankton on the filter material and the mesh size of the plankton net, likely not capturing all diatoms. Nanoscale secondary ion mass spectrometry (nanoSIMS) analysis was performed using a NanoSIMS 50 L (CAMECA, Paris, France) as previously described [26]. Detailed descriptions can be found in the Suppl. Methods.

### Multivariate statistics

Principle components analysis (PCA) was performed using the R package factoMineR’ 2.112.5 [52] for samples collected in the South Pacific. All samples for which all metadata (N_2_ fixation rate, primary production rate, chl *a*, nutrient concentrations, temperature, and salinity) were available were included for analysis. When the sampling depth deviated more than 5 m from the depth of the incubations, nutrient, chl *a* concentrations and temperatures were linearly interpolated. This interpolation was done for nine of the 38 samples, and maximum deviation was ~12 m. About half of the interpolations were done for samples from the chl *a* maximum or deeper. Explanatory variables were standardized (z-scoring) prior to analysis.

### Data visualization

Data was visualized in R [53] using the packages “ggplot2” [54], “maps” [55], “phyloseq” 1.29 [56] and “factoextra” 1.0.7 [57] as well as GraphPad Prism, look@nanoSIMS [58] and Ocean Data View [59].

## Results

### South Pacific Ocean chemistry and N_2_ fixation

Our cruise to the South Pacific covered two distinct oceanographic regimes (Fig. 1): the oligotrophic waters of the central South Pacific Gyre (Stations 2–10) with its low surface chl *a* concentration (Fig. 1A) and the mesotrophic Southwest Pacific with moderate chl *a* concentrations (Stations 11 to 15). Surface waters in the gyre showed a depletion in NO_3_^−^ and NO_2_^−^ (hereafter, NO_x_^−^) (Fig. 1B), with NO_x_^−^ concentrations <0.2 μmol L^−1^ above the nutricline, which was located at 150–200 m depth in the core of the gyre. The nutricline shoaled towards the gyre rim at both the Eastern border (Stations 2 to 1) and near the Subtropical South Pacific Front southwest of Station 10. Low but persistent N_2_ fixation rates, ranging from 0.1 (± 0.1) - 0.8 (± 0.1) nmol N L^−1^ d^−1^, were measured in the South Pacific Gyre, with detectable rates down to a maximum depth of 200 m at Station 6 (Fig. 1C). In the mesotrophic Southwest Pacific, N_2_ fixation rates were lower than in the gyre (<0.3 nmol N L^−1^ d^−1^) and were generally restricted to waters between 20 and 100 m depth. N_2_ fixation activity was not detected at Station 1 at the eastern edge of the gyre and at Station 12 in the mesotrophic Southwest Pacific. At Station 12, the lack of N_2_ fixation activity coincided with a local intrusion of waters with NO_x_^−^ concentrations >1 μmol L^−1^ to the surface (Fig. 1B). This intrusion was located closely to the South Pacific subtropical front where nutrient-rich, subantarctic waters of the Southern Ocean commonly mix with nutrient-poor waters of the subtropical South Pacific gyre [60]. Overall, measured N_2_ fixation rates were similar to previous reports from the subtropical South Pacific gyre [13, 30, 61], and broadly overlapped with the NO_x_^−^-depleted surface waters between 80 and 130 degrees West (Fig. 1B and C).

### Diversity and distribution of *nifH* phylotypes

**Figure 2:**
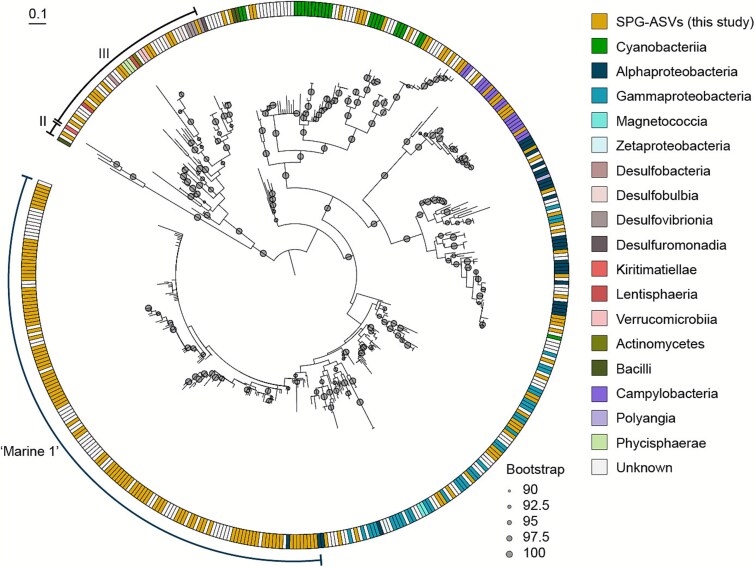
Amino acid-based phylogenetic tree of NifH sequences showing the placement of the 174 ASVs from the South Pacific gyre (SPG-ASVs). The taxonomy of each sequence is indicated by color codes on the right and is based on class-level GTDB taxonomy. Reference sequences without a known taxonomy are designated as “unknown”. Bootstrap values from 90 to 100% are indicated with grey dots. The canonical *nifH* clusters II and III as well as the group “Marine 1” are indicated by curved brackets. Accession numbers of reference sequences can be found in the tree file (Suppl. Data File 4).

Amplicon analysis of all partial *nifH* sequences resulted in 174 unique *nifH* ASVs belonging to groups I, II and III of the six described *nifH* groups [62]. While only a few ASVs (14) with low relative abundance were assigned to clusters II (1) and III (13), most of the ASVs (160) fell into *nifH* cluster I (Fig. 2) [63]. Within cluster I, two ASVs were associated with *Trichodesmium*, seven ASVs were associated with UCYN-A, and another six ASVs were associated with either the UCYN groups B/C (two ASVs) or filamentous heterocystous cyanobacteria (four ASVs). These cyanobacterial sequences were however, present at low relative abundances and were restricted to a few stations and depths (Suppl. Fig. 1). Another 43 of the 160 cluster-I ASVs grouped with diverse sequences mostly from Gamma- and Alphaproteobacteria as well as Campylobacteria (Fig. 2).

**Figure 3:**
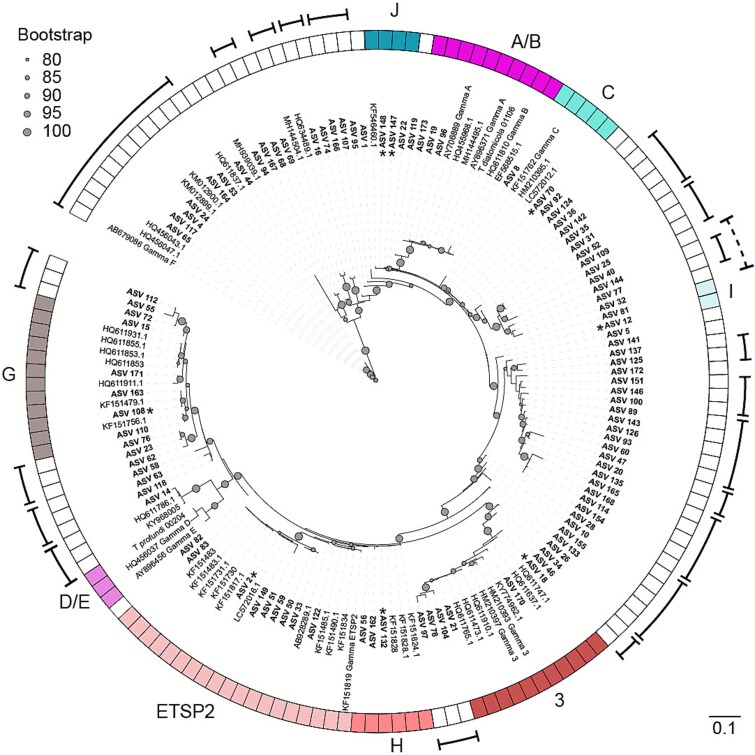
Pruned nucleotide-based phylogenetic tree of *nifH* sequences that affiliated with the members of the group “Marine 1” showing in color the clades containing either of the two *Ca*. Tectiglobus species or any of the 10 most abundant ASVs across the whole dataset (with clades named after their reference sequence (e.g. ETSP2) as summarized by Langlois *et al.* [22] or their new clade name, e.g. G). Other clades are indicated by curved brackets. ASVs from this study are shown in bold while reference sequences are in regular font. Bootstrap support from 80 to 100% is shown by grey dots. ASVs belonging to the overall 10 most abundant ASVs are indicated by asterisks on the inside of the leaf labels. Accession numbers are given as leaf labels and can also be found in the accompanying tree file (Suppl. Data File 3).

The majority of the ASVs (102 ASVs, i.e. 64%) belonging to *nifH* cluster I grouped (bootstrap support 89.3%) with *nifH* sequences that lacked cultured representatives and have previously been found in the eastern tropical North Pacific [17] and Atlantic [64], South Pacific Gyre [13], the Eastern tropical South and North Pacific [17, 46], the Eastern tropical North Atlantic [64], the South China Sea [65, 66] and the Indian Ocean [16] (Fig. 2). Sequences within this group were previously reported as Gamma-A to -F, Gamma-ETSP2 (γETSP2) or Gamma-3 phylotypes [13, 22, 46], collectively termed group “Marine 1” [22]. This group also contained the *nifH* sequences of both *Ca*. *T. diatomicola* and *Ca*. *T. profundi* [26]. *Ca*. *T. diatomicola* is an alphaproteobacterial diatom symbiont that obtained its capacity to fix N_2_ through horizontal transfer of *nif* genes from a gammaproteobacterium. This *nifH* phylotype was hence previously termed “Gamma-A”. *Ca*. *T. profundi*, the closest relative of Ca. *T. diatomicola* and also an alphaproteobacterium within the Rhizobiales, is likely also a symbiont given its small genome and the reduced GC content ([26]; also see methods for genome refinement). It has, however, so far not been visualized and a symbiotic interaction has not been shown. Based on the position of the two *Ca*. Tectiglobus sequences (Figs 2 and 3), we consider sequences that shared a common node with the Ca. Tectiglobus sequences as relatives of these two organisms that may form distinct clades. Analogous to the clades of the UCYN-A-haptophyte symbioses, we therefore applied a clustering analysis at the nucleotide level that resulted in 30 clades, some of which appeared to be paraphyletic while many consisted of only two or three sequences (Fig. 3). In addition, several ASVs (12) did not fall into any clade possibly due to a lack of representative sequences. Besides the phylotype Gamma-F, all Gamma phylotypes previously summarized by Langlois *et al.* [22] as “Marine 1” shared a common node with the two *Ca*. Tectiglobus species (bootstrap support 98.3%) with *Ca*. *T. diatomicola* located in clade A/B and *Ca*. *T. profundi* located in clade D/E (Fig. 3). Although many ASVs grouped with “Marine 1” members, only few clades contained ASVs that were abundant. Further analysis therefore focused on clades containing abundant ASVs as well as the two clades containing the *Ca*. Tectiglobus species. This subset consisted of nine clades (A/B, C, D/E, 3, ETSP2 and the new clades G, H, I, and J) and accommodated eight of the 10 most abundant ASVs in the data set (Fig. 3). Sequences within each clade shared between 95.6 and 99.7% sequence similarity (cutoff of 95% was used for analysis; see methods) at the nucleotide level and on average 88.5% similarity across all nine clades (Suppl. Fig. 2). Given that clade A/B and clade D/E accommodate the two alphaproteobacterial *Ca*. Tectiglobus species, there is considerable doubt regarding the taxonomic assignment of the clades in group “Marine 1”. We hence refrain from referring to the clades in group “Marine 1” as “Gamma” phylotypes. Instead, we now refer to these clades as ‘Marine 1-A/B, Marine 1-C, etc. (Fig. 3) with the exception of gamma-F which we do not consider part of the “Marine 1” group for the purposes of this manuscript.

**Figure 4:**
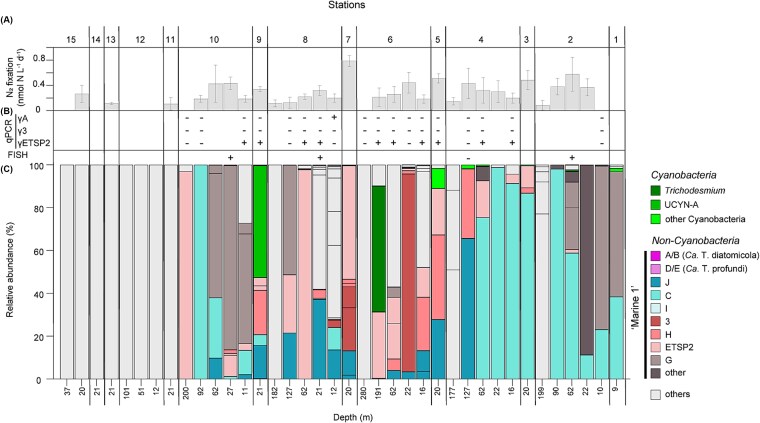
Relative abundance of *nifH* ASVs and their phylogenetic affiliation at individual stations and depths throughout the South Pacific gyre (C) along with the respective N_2_ fixation rates (A) and detection of clades by qPCR assays (γA, GammaA; γ3, Gamma3; and γETSP2) and *Ca*. Tectiglobus-diatom symbioses by FISH (B) showing the prevalence of “Marine 1” clades in the central gyre region. Each column represents an individual sample from a different depth (number at bottom) at a different station (number at top). In (A), error bars indicate the standard deviation of triplicate incubations and missing bars indicate rates below the detection limit. Each stack in (C) represents an individual *nifH* ASV. Note that only 38 out of 49 samples are displayed, as N_2_ fixation activity was not determined in the additional 11 samples that were sequenced. Those 11 samples are, however, included in the overall analysis and are also displayed in Suppl. Fig. 1. Plus and minus signs in panel (B) indicate samples in which we did (+) or did not (−) detect diatoms containing FISH-positive cells (also see Table 1) or *nifH* copies using qPCR.

The designated “Marine 1” clades were not evenly distributed across the South Pacific based on relative abundances (Fig. 4 and Suppl. Figs 1 and 3). Clade C and clade G with the two most abundant ASVs were present mostly at the eastern and western edge of the gyre where they comprised on average ~50% and up to 100% of the total *nifH* reads when present. Clusters 3, H, and I were located more toward the center of the gyre where they comprised on average ~40%, ~15% and ~25%, respectively, of the *nifH* reads when present (Suppl. Fig. 1). Members of clade ETSP2 (containing the third most abundant ASV) were mostly present in the center of the gyre and at the southwestern edge, where it comprised on average ~20% of the total *nifH* reads (Suppl. Fig. 1). The clade A/B, containing *Ca*. Tectiglobus diatomicola, was only present at two stations in the center of the gyre with a maximum relative abundance of 0.3% of the total *nifH* reads. Clade D/E, containing *Ca*. Tectiglobus profundi, was not detected in any of our samples. Overall, “Marine 1” clades were found almost exclusively in waters with observable N_2_ fixation rates, where they often constituted more than 90% of the total *nifH* reads (Fig. 1d).

Several phylotypes within the “Marine 1” clades have previously been quantified as *nifH* copies using qPCR. We used previously published assays for the phylotypes GammaA ([37]; clade Marine 1-A/B), Gamma3 ([13]; clade Marine 1–3) and γETSP2 ([46]; clade Marine 1-ETSP2) to provide quantitative context for the relative abundances determined here. While we could not detect any *nifH* copies with the Gamma3 assay, the GammaA assay detected *nifH* copies only in the sample where ASV19 of clade A/B was also detected through *nifH* amplicon sequencing (Fig. 4). The γETSP2 assay detected *nifH* copies in various samples, in almost all of which we also detected ASVs of the clade ETSP2 through sequencing. For both the GammaA and γETSP2 assays, *nifH* copies were detectable but not quantifiable (i.e. less than 120–180 *nifH* copies L^−1^).

### Visualization, abundances, and single-cell activities of *Ca.* Tectiglobus spp.

In order to assess the specificity of our FISH assay, we recovered the full-length 16S rRNA gene sequence of *Ca*. *T. profundi* by genome refinement (see Methods). In silico analysis showed that the FISH probes used here target both *Ca*. *T. diatomicola* and *Ca*. *T. profundi* species (Suppl. Fig. 4). To visualize *Ca*. Tectiglobus spp. in the South Pacific gyre, we carried out FISH on plankton net samples and selected samples from discrete depths at five stations spanning the gyre region (Fig. 1 and Table 1).

**Table 1:** Presence of *Ca*. Tectiglobus-diatom symbioses in plankton net and depth-specific samples from five investigated stations. n.a. not available, n.d. not detected.

Station	Plankton net depth (m)	Detected in net	Specific depth (m)	Bulk N_2_ fixation (nmol N L^−1^ d^−1^)	Abundance (symb. L^−1^)	Contribution of symbioses^a^ (nmol N L^−1^ d^−1^)
SO245–2 23.5°S 90.0°W	0–250	yes	62	0.58 ± 0.22 (62 m)	128	0.14
SO245–4 23.5°S 100°W	0–250	yes	127	0.43 ± 0.20 (127 m)	n.d.	-
SO245–6 23.5°S 110.0°W	0–280	yes	n.a.	-	-	-
SO245–8 27.7°S 117.6°W	0–300	yes	21	0.32 ± 0.06	107	0.11
SO245–10 33.5°S 126.0°W	0–300	yes	27	0.43 ± 0.08	160	0.17

a Based on average single-cell rate for symbiosis (1058 fmol N symb.^−1^ d^−1^) from all four measured diatoms at stations 2, 8, and 10.

**Figure 5:**
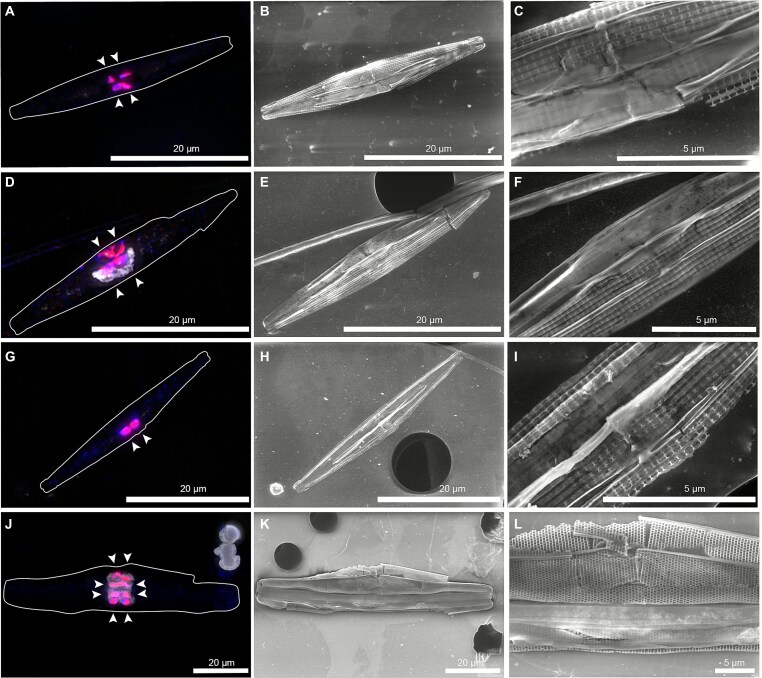
Correlative epifluorescence (A, D, G, J) and scanning electron (B, E, H, K) microscopic imaging of four diatom hosts with their *Ca*. Tectiglobus symbionts (shown in pink as overlay of Hypho638–Hypho825 mix in blue and Hypho1147 in red, respectively) identified in the South Pacific gyre after FISH; nucleic acids stained with DAPI shown in white. Panels C, F, I. and L show zoom-ins of the central symbiont-containing region of the diatoms shown in B, E, H, and K. The majority of diatom hosts (A–I) were morphologically similar to those identified in the tropical North Atlantic [26], while one diatom (J–L) found at station 8 differed morphologically. This diatom contained eight symbionts likely due to ongoing cell division. Note the different scale bars. Arrowheads point toward individual FISH-positive cells (also see Suppl. Fig. 5). Diatom hosts are outlined in A, D, G, and J.

We detected FISH-positive cells that were located inside pennate diatoms at all five investigated stations, including samples where clade A/B – to which *Ca*. *T. diatomicola* belongs—and clade D/E—to which *Ca*. *T. profundi* belongs—had a very low relative abundance or were entirely absent (Fig. 4 and Suppl. Fig. 1). Of the 78 diatoms imaged by fluorescence microscopy and SEM, 36 diatoms contained four centrally located symbionts and 36 diatoms contained two centrally located symbionts (Fig. 5 and Suppl. Fig. 5). This differs from the *Ca*. *T. diatomicola*-*Haslea* symbioses in the tropical North Atlantic, which typically contained four centrally located symbionts. Just like for the tropical North Atlantic, diatom symbioses with six or eight symbionts were observed that represent symbioses at different stages of cell division (Fig. 5 and Suppl. Fig. 5) [26]. The remaining diatoms containing either three or one centrally located symbiont(s), represent broken diatoms or diatoms oriented in such a way (girdle view, valve view or an intermediary view) that symbionts could not be properly visualized.

Most of the diatom hosts were found to be morphologically similar to those identified in the tropical North Atlantic [26] with cell lengths of 30–44 μm (mean ± SD of 36.9 ± 3.2 μm; n = 53) and cell widths of 3.6–8.2 μm (mean ± SD of 5.0 ± 0.9 μm; n = 53) (Fig. 5). One diatom found in the plankton net sample from station 8 was substantially larger (85 μm long and 14.5 μm wide) and morphologically different (Fig. 5j-l). This diatom contained eight symbionts, likely as a result of active cell division [26]. Its gross frustule morphology exhibited some differences to the host diatom described in Tschitschko *et al.* [26] with the valve edge tapering more strongly towards a narrow valve pole. Moreover, the longitudinal strips overlaying the striae externally in the genus *Haslea* were also absent. Since only one frustule of this type was found and only the exterior of the valve was visible, an unambiguous assignment to a genus cannot be made at this stage.

**Figure 6:**
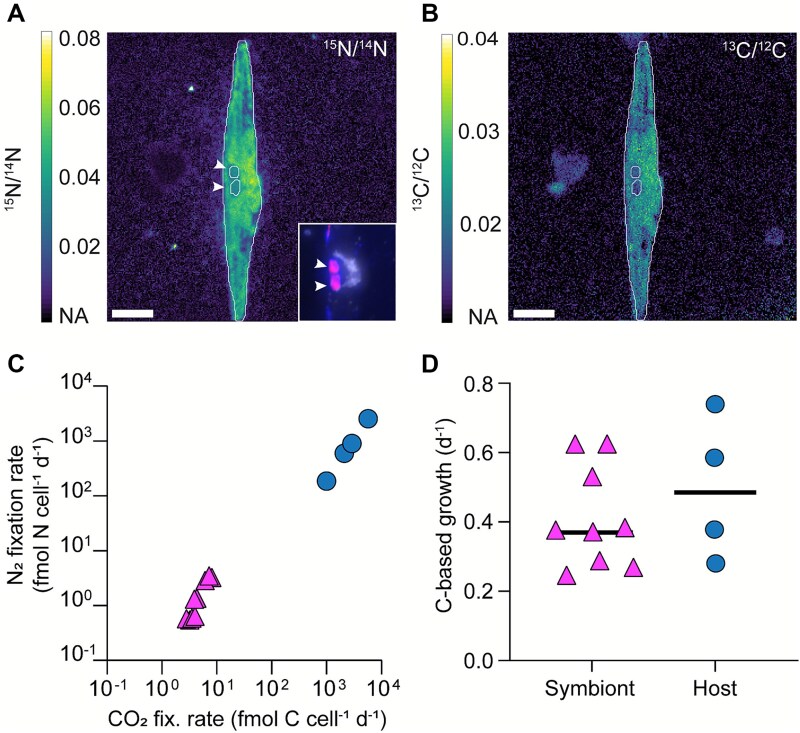
NanoSIMS single-cell imaging of enrichment in ^15^N from ^15^N_2_ fixation (A) and ^13^C enrichment from ^13^CO_2_ fixation (B); the inset shows the respective fluorescence image after hybridization of *Ca*. Tectiglobus symbionts (indicated by white arrowheads) with specific oligonucleotide probes (in pink, overlay of Hypho638–Hypho825 mix in blue and Hypho1147 in red, respectively). Corresponding single-cell rates of *Ca.* Tectiglobus (triangles, pink, *n* = 9) and their host diatoms (circles, blue, *n* = 4) (C) and their carbon-based growth rates (d; black lines indicate means) obtained from stations 2, 8, and 10.

Absolute abundances based on microscopic counts of diatoms containing symbionts (FISH-positive cells) ranged from 107 to 160 diatoms L^−1^ in depth-specific samples with the exception of Station 4 where we detected *Ca.* Tectiglobus signals only in plankton net samples (Table 1). Single-cell isotope ratio measurements of four diatoms from three different samples of the gyre region revealed that all measured diatoms and their symbionts were enriched in ^15^N and ^13^C from the fixation of ^15^N-enriched N_2_ and ^13^C-enriched CO_2_, respectively (Fig. 6). Based on the limited number of measurements from the South Pacific, the single-cell N_2_ fixation rates of 186 to 2552 fmol N symbiosis^−1^ d^−1^ (average ~1058 fmol N symbiosis^−1^ d^−1^; Fig. 6, Table 1) are comparable to those measured in the tropical North Atlantic [26]. Carbon-based growth rates were somewhat lower (~0.5 d^−1^) than those determined in the tropical North Atlantic (0.8 d^−1^) (Fig. 6). Combining the abundances with the single-cell rates showed that these symbioses were responsible for ~25–40% of the measured bulk N_2_ fixation rates in the South Pacific Gyre (Table 1).

### Ecophysiology of the “Marine 1” clades

To gain insight into the ecophysiology of members of the “Marine 1” group, individual samples were ordinated using PCA based on the samples’ measured environmental parameters (Suppl. Fig. 6). The first two principal components (PCs) explained 71% of the variance in the data set. PC1 was negatively correlated with primary production, chl *a* concentrations and nutrients, and positively correlated with N_2_ fixation, temperature and salinity. PC2 was positively associated with the nutrient concentrations and was negatively correlated with primary production and chl *a* concentrations. Samples with a higher relative abundance of “Marine 1” *nifH* sequences clustered with temperature, salinity and N_2_ fixation and mainly originated from the photic zone of the gyre while samples from deeper and/or more nutrient-rich waters showed the lowest “Marine 1” abundances (Suppl. Fig. 6).

## Discussion

Along the ~8000 km transect through the subtropical South Pacific, N_2_ fixation was most prevalent in the gyre waters (Stations 2–10) with rates similar to previous observations [13, 30, 67]. The lack of detectable N_2_ fixation on the eastern margin is largely in agreement with measurements of N_2_ fixation in the vicinity or directly above oxygen minimum zones where nitrogen loss occurs [68]. Combined with the lack of N_2_ fixation at Station 12 in the Southwest Pacific, likely caused by the intrusion of nitrate-rich waters, this result indicates that the N_2_-fixing microbial community in the South Pacific might be relatively sensitive to dissolved inorganic nitrogen. Cyanobacterial N_2_-fixers were relatively rare or undetectable in the South Pacific gyre and had a low diversity, consistent with previous reports for this region [13, 30, 46, 69]. Instead, based on relative abundances, the N_2_-fixing community was dominated by non-cyanobacterial *nifH* phylotypes, indicating that non-cyanobacterial N_2_-fixers play a major role in the South Pacific gyre. Yet, neither non-cyanobacterial nor cyanobacterial N_2_-fixers have been shown to actively contribute to N_2_ fixation in this region, the largest surface water biome on our planet.

Our phylogenetic analyses revealed that the majority of the non-cyanobacterial *nifH* ASVs fell into the uncultured group “Marine 1”. This group harbors the widespread and globally abundant phylotype Gamma-A [22], which was recently identified as a heterotrophic alphaproteobacterial diatom symbiont, *Ca*. Tectiglobus diatomicola [26]. Its closest relative, the alphaproteobacterial *Ca*. *T. profundi*, likely also a symbiont [26], is also part of the “Marine 1” group and clusters with the phylotypes Gamma-D and Gamma-E, now designated as “Marine 1-D/E”. Both *Ca*. Tectiglobus species have previously been detected in the South Pacific gyre through metagenomic mapping [26]. Using FISH targeting both known *Ca*. Tectiglobus species, we observed pennate diatoms containing FISH-positive cells at all investigated stations throughout the South Pacific gyre. Intriguingly, neither of the two clades containing the known *Ca*. Tectiglobus species was present in four of the five investigated stations based on *nifH* amplicon sequencing. At a single depth of the fifth station, clade A/B was at only 0.14% relative abundance with detectable but not quantifiable *nifH* copies using the GammaA assay. This conundrum indicates that our FISH approach is targeting close relatives of the two known *Ca*. Tectiglobus species and that these relatives likely belong to the *nifH* phylotypes most closely related to the *Ca*. Tectiglobus *nifH* sequences, namely other phylotypes of group “Marine 1”. At this stage, however, we cannot link our microscopic imaging to a distinct clade within the “Marine 1” group due to the lack of information of their 16S rRNA gene sequences that may be resolved in future metagenomic sequencing and isolation efforts. Nevertheless, our combined FISH, SEM and nanoSIMS results show that at least some of these relatives in the group “Marine 1” are also diatom symbionts that actively fixed N_2_ in the South Pacific gyre. To our knowledge, this is the first confirmed N_2_-fixer actively contributing to N_2_ fixation in this region.

Relatives targeted by the *Ca*. Tectiglobus FISH probes associated with at least two different host diatoms in the South Pacific gyre. Most of the host diatoms had a morphology similar to the *Ca*. *T. diatomicola* host in the tropical North Atlantic [26] and likely harbor members of the clades with higher relative abundances (clades C, G, ETSP2, or J). Using qPCR, we detected *nifH* copies with the γETSP2 assay; however, the *nifH* copy numbers were lower than what would be expected based on our direct counts of the diatom symbioses. Assuming a minimum of two symbionts per diatom, those abundances (107 to 160 diatoms L^−1^) would result in *nifH* copy numbers in the order of 2–3 × 10^2^  *nifH* copies L^−1^, only marginally higher than our qPCR quantification limit (1.2–1.8 × 10^2^ copies L^−1^). The slight discrepancy between estimated *nifH* copy numbers (based on direct microscopic counts) and *nifH* copies from qPCR implies that phylotypes other than clades A/B, 3 and ETSP2 harbor diatom symbionts. The clade ETSP2 has previously been detected via qPCR in the South Pacific [46] with abundances of up to ~3 × 10^2^  *nifH* copies per L indicating that absolute abundances of this phylotype can vary in space and time. The lack of detectable *nifH* copies from the clade Marine 1–3 is consistent with the original report in the South Pacific gyre [13]. However, it has previously been detected in the North Pacific subtropical gyre with abundances of up to ~7 × 10^3^  *nifH* copies L^−1^ [70] in waters that typically exhibit higher N_2_ fixation rates than the South Pacific gyre [11]. Their recurrent detection in the >10 μm size fraction is consistent with the idea that this clade harbors symbionts [70]. Although we cannot directly link a specific clade to the morphologically different diatom found at station 8, we presume that this diatom likely hosts one of the rarer “Marine 1” members in the South Pacific gyre because we found only a single such diatom in our samples. UCYN-A symbionts also associate with differently sized but closely related hosts [48, 71] that have different growth rates [9]. We can only speculate that the “Marine 1” clade member associated with the larger diatom may also exhibit distinct physiological traits that impact their cellular activities and their relevance for the marine carbon and nitrogen cycles.

Interestingly, the distribution and relative abundance of individual clades of the “Marine 1” group varied throughout the South Pacific. Some clades were found throughout most of the gyre, while others appeared to be more region-specific indicating potential differences in their ecophysiology. It is likely that these clades, related to *Ca*. *T. diatomicola* and *Ca. T. profundi*, are also symbionts of pennate diatoms, and as such their distribution patterns would remind of the ecotypes found for the well-known non-N_2_-fixing cyanobacteria *Prochlorococcus* [72] and *Synechococcus* [73, 74] as well as for the N_2_-fixing cyanobacterial symbiont UCYN-A [75]. To date, eight different sublineages of UCYN-A have been identified of which some appear more prevalent in the open ocean whereas others seem to be more prevalent in marginal seas or coastal areas [48, 75]. Just like *Prochlorococcus* and *Synechococcus* ecotypes [76, 77], occurrences of UCYN-A ecotypes may be modulated by environmental parameters [78]. While higher concentrations of dissolved inorganic nitrogen are often presumed to inhibit N_2_ fixation activity and drive distribution patterns, some UCYN-A symbioses appear to maintain N_2_ fixation also in nitrate-rich waters [79]. In the South Pacific gyre, concentrations of dissolved inorganic nitrogen >0.5 μM appear to inhibit N_2_ fixation activity as well as the growth of “Marine 1” group members. Other, yet unknown parameters likely also impact members of the different clades as their distribution varied throughout the nitrate-depleted South Pacific gyre.

Our results show that the group “Marine 1” contains several other close relatives of *Ca. T. diatomicola* and *Ca*. *T. profundi* that are also N_2_-fixing symbionts of pennate diatoms expanding the diversity of both symbionts and hosts. The association with diatom hosts would also explain why *nifH* genes belonging to “Marine 1” members have previously been found in larger size fractions [46]. In general, gammaproteobacterial *nifH* sequences are frequently detected in larger-size fractions or marine particles (e.g. [80, 81]), and although many of these may in fact be alphaproteobacterial *Ca*. Tectiglobus symbionts, some may indeed be “bonafide” gammaproteobacteria [24] that actively fix N_2_ [82]. It has so far been difficult to estimate the contribution of these and other non-cyanobacterial N_2_-fixers to oceanic nitrogen fixation [82]. Based on our results, the *Ca*. Tectiglobus-diatom symbioses alone could be responsible for up to ~40% of N_2_ fixation in the South Pacific gyre. Given the size of this largest oceanic biome with a surface area about three times as large as the North Atlantic gyre and the abundance of non-cyanobacterial *nifH* genes in other ocean regions, heterotrophic N_2_-fixers likely play a major role in marine nitrogen cycling.

## Supplementary Material

Supplementary_Figures_and_Tables_ver2_ycaf207

Supplementary_Methods_ver2_ycaf207

SupplDataFile1_LOD_MQR_ycaf207

SupplDataFile2_ASVs_ycaf207

SupplDataFile3_NT_nifH_ycaf207

SupplDataFile4_AA_NifH_ycaf207

SupplDataFile5_qPCR_mismatch_table_ycaf207

SupplDataFile6_T_profundi_16S_ycaf207

## Data Availability

The *nifH* sequencing data has been deposited at the European Nucleotide Archive (ENA) under the Bioproject number PRJEB92104, and the ASV sequences are available in Suppl. Data File 2. The 16S rRNA gene sequence of *Ca*. Tectiglobus profundi is available in Suppl. Data File 6. Tree files for both the nucleotide-based and amino acid-based trees can be found as Suppl. Data Files 3 and 4.
