# Supplementary material for: Diverse community of rhizobia-diatom symbioses fixes nitrogen in the South Pacific gyre
Source: ISME Commun. 2025 Nov 12;5(1):ycaf207. doi: 10.1093/ismeco/ycaf207 (PMC12667272; doi:10.1093/ismeco/ycaf207)
Supplement: Supplementary_Figures_and_Tables_ver2_ycaf207 [file supplementary_figures_and_tables_ver2_ycaf207.docx]

**Supplementary Figures and Tables**

**to**

**Diverse community of** **Rhizobia-diatom symbioses fixes nitrogen in the South Pacific Gyre**

Mertcan Esti^1,‡^, Miriam Philippi^1,4,‡^, Julia Duerschlag^1,5^, Timothy G. Ferdelman^1^, Jennifer Tolman^2^, Julie LaRoche^2^, Clara Martínez-Pérez^1,6^, Gaute Lavik^1^, Bernhard Tschitschko^1,7^, Hon Lun Wong^1^, Alexandra Kraberg^3^, Sten Littmann^1^, Abiel T. Kidane^1^, Wiebke Mohr^1,*^, Marcel M. M. Kuypers^1^


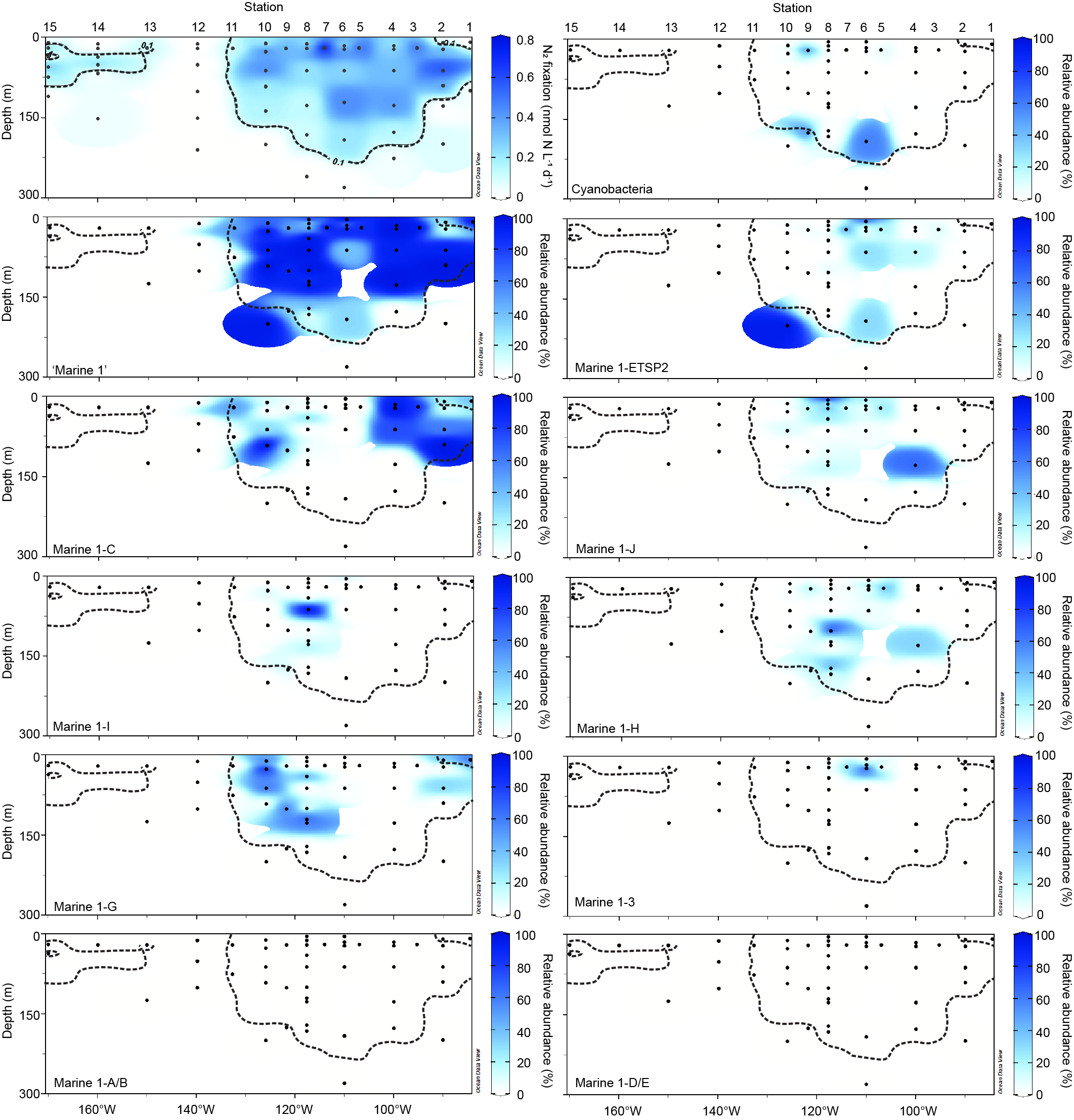


**Supplementary Figure 1** Distribution of N_2_ fixation rates, cyanobacteria and individual ‘Marine 1’ clades (as % relative abundance) across the cruise transect and with depth. Dashed lines indicate the isoline of an N_2_ fixation rate of 0.1 nmol N L^-1^ d^-1^.


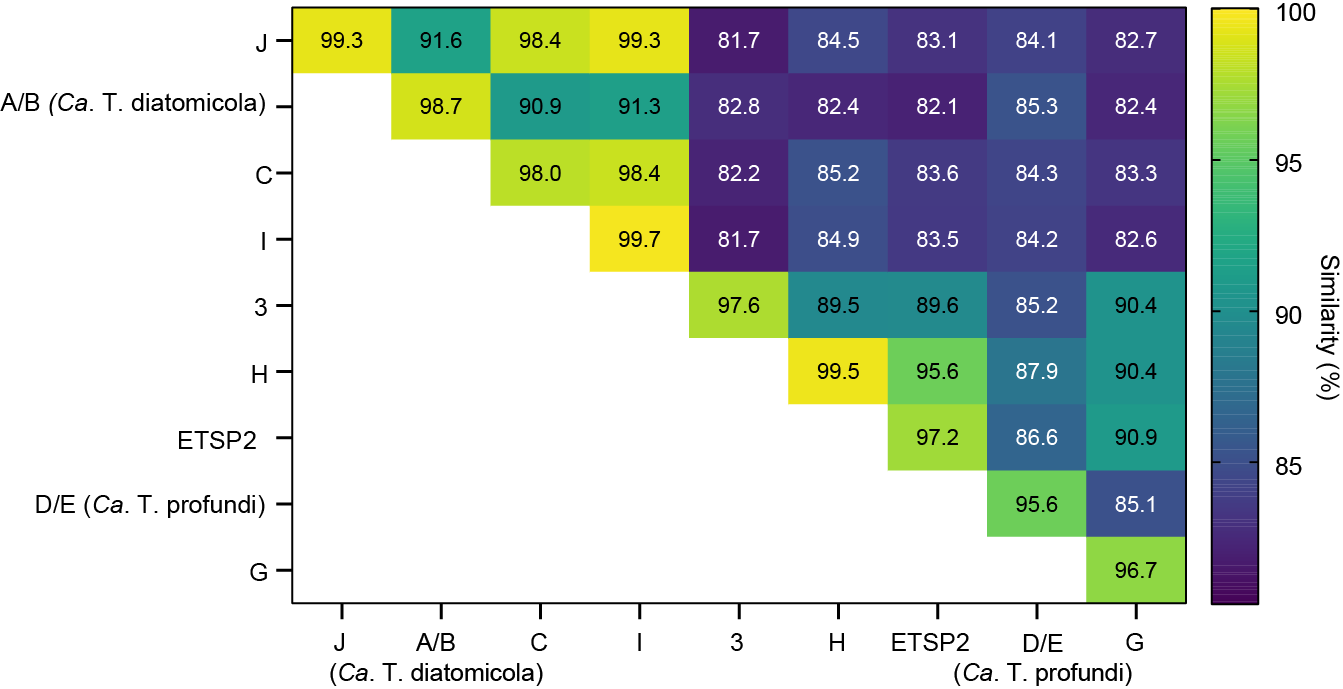


**Supplementary Figure 2** Similarities of *nifH* sequences within and between the different clades of group ‘Marine 1’ using pairwise comparison. Clades are shown in the order they appear in the tree (Fig. 3).


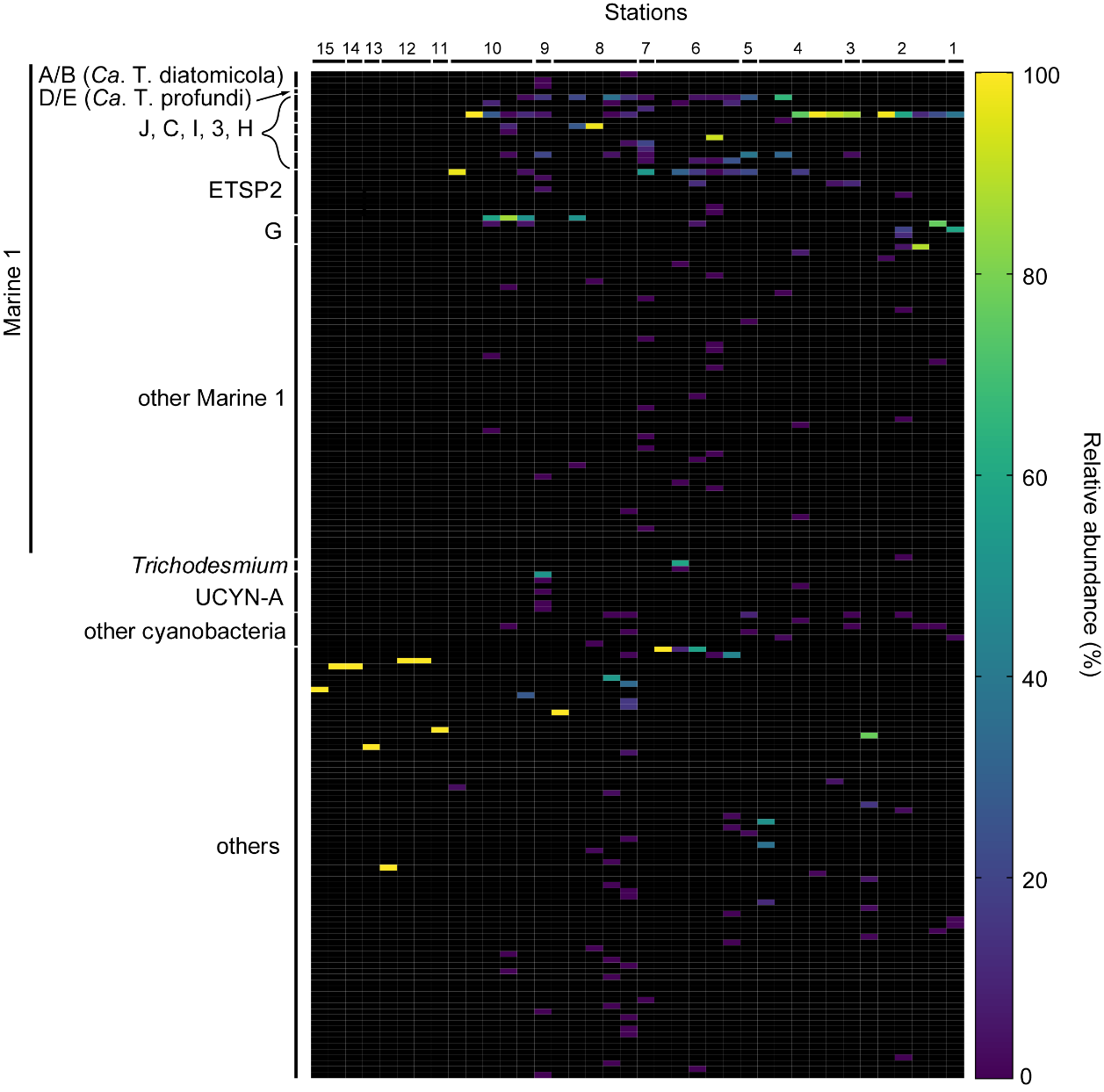


**Supplementary Figure 3** Relative abundance of the 174 recovered *nifH* ASVs throughout the South Pacific by stations and depths, aligned as in Figure 4. The phylogenetic affiliation of the *nifH* ASVs is indicated on the left.


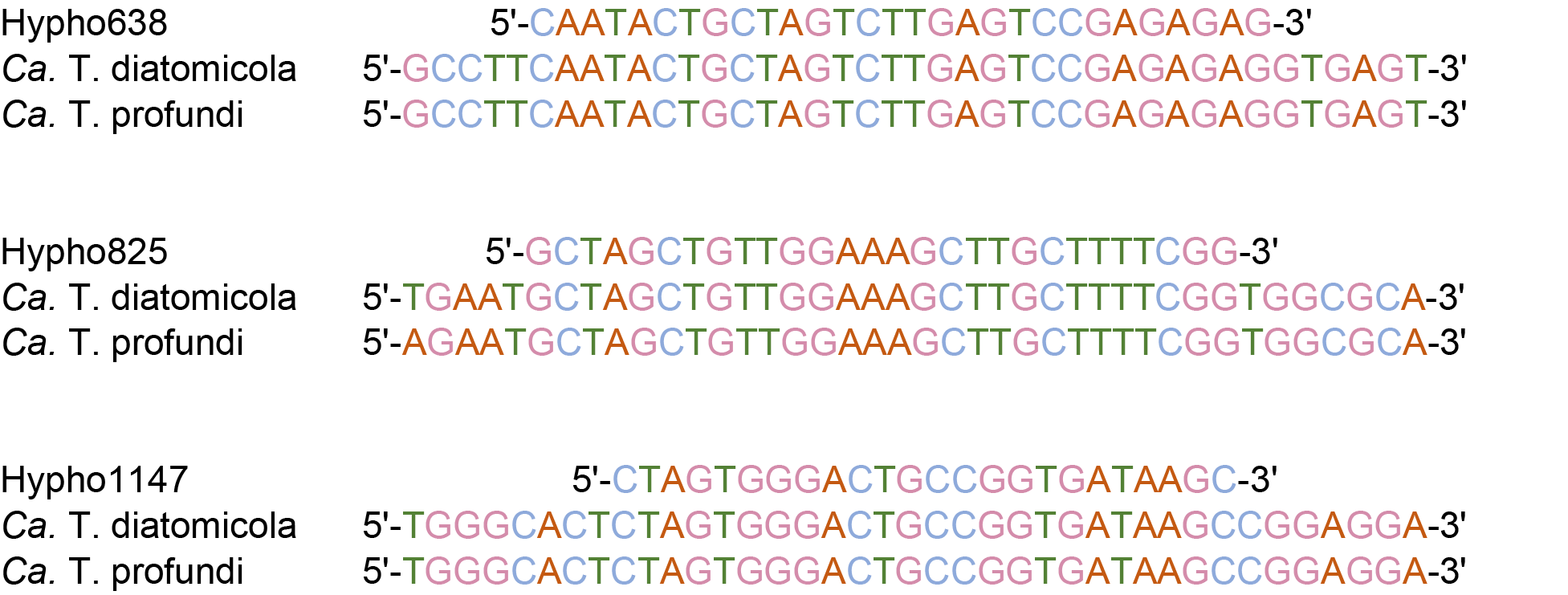


**Supplementary Figure 4** Alignment of 16S rRNA gene sequences of *Ca*. Tectiglobus diatomicola and *Ca*. T. profundi with FISH probes used in this study showing that both species are targeted by the three probes without any mismatches.


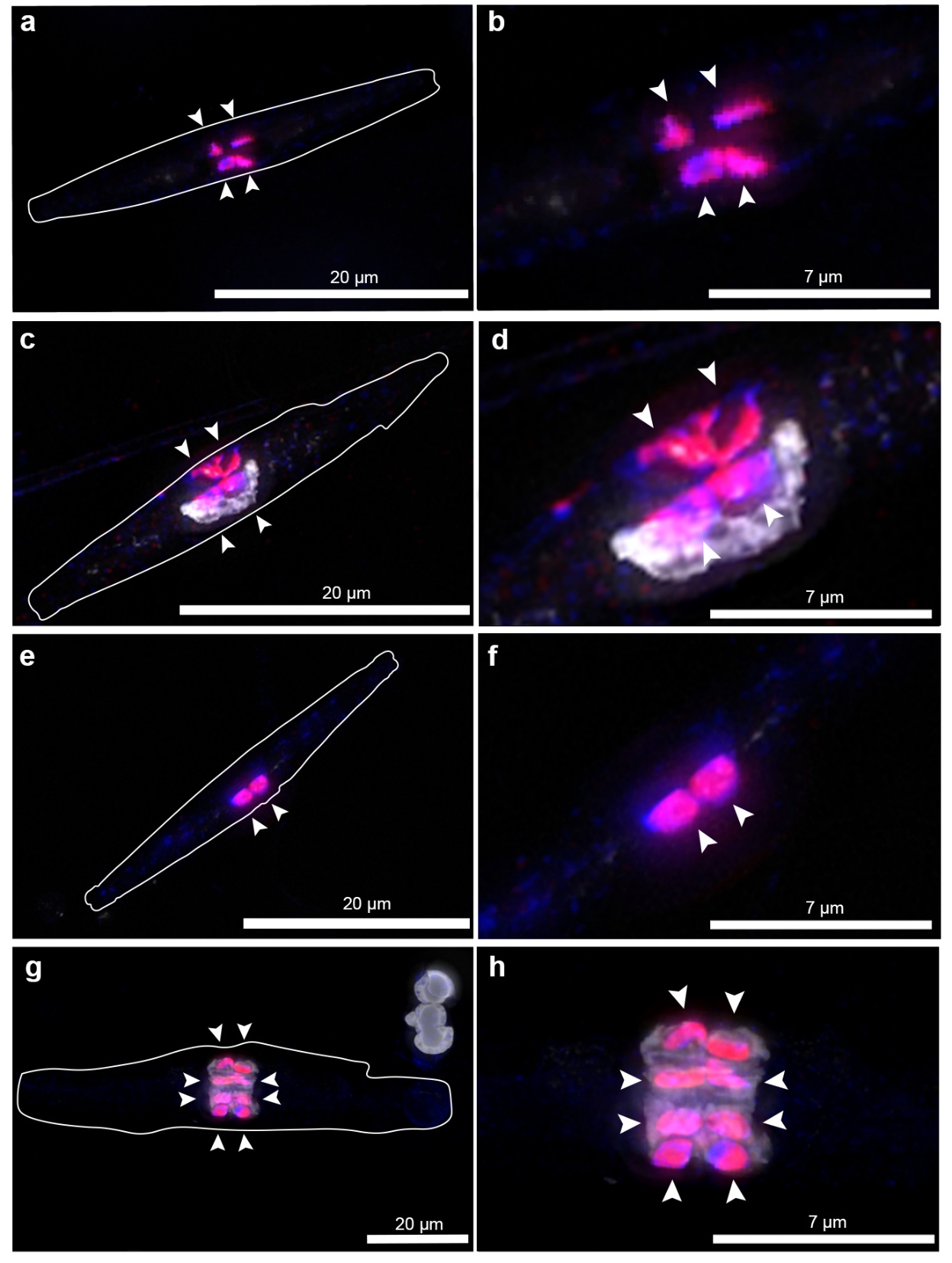


**Supplementary Figure 5** FISH images of diatoms in Fig. 5 (a, c, e, g) and corresponding zoom-in images of the centrally-located symbionts to better visualize the number of symbionts (b, d, f, h). Symbionts (indicated by arrowheads) are shown in pink as overlay of Hypho638–Hypho825 mix in blue and Hypho1147 in red, respectively; nucleic acids stained with DAPI shown in white. Respective diatom hosts are outlined in a, c, e, and g. The morphologically different diatom in panels g and contained eight symbionts likely due to ongoing cell division.


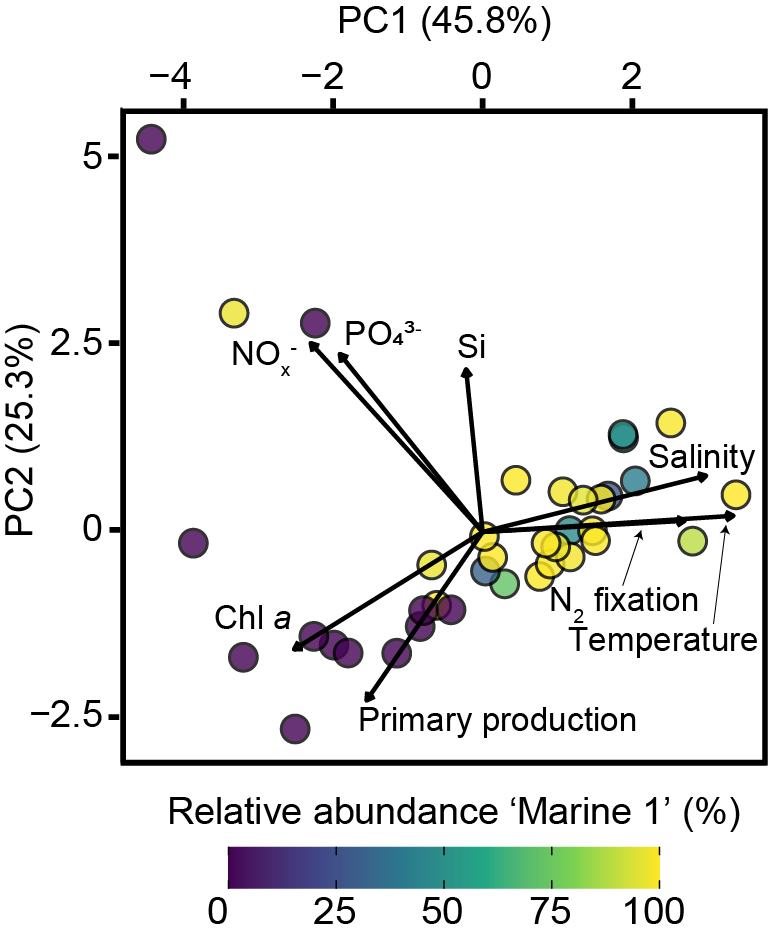


**Supplementary Figure 6** Principal component analysis of the South Pacific samples depicting the differences between the individual samples (circles) based on the given environmental parameters. Color coding represents the relative abundance of sequences belonging to the ‘Marine 1’ group.

**Supplementary Table 1:** Forward (F) and reverse (R) primers and probe sequences of qPCR assays used in this study and mismatches to selected representative sequences from target clades. Asterisks indicate ASVs belonging to the ten most abundant ASVs in the dataset. Please note that the bold and underlined nucleotide positions in the reverse primer 2 (R2) of the Gamma3 assay differ from the original assay (Halm et al. 2012 [13]).

| **Assay** | **Primer/**  **probe** | **Sequence (5’>3’)** | **Mismatches to clades** | | | | |
| --- | --- | --- | --- | --- | --- | --- | --- |
|  |  |  | **A/B** | **3** | | **ETSP2** | **H** |
|  |  |  | **ASV**  **19** | **ASV**  **18*** | **ASV**  **21** | **ASV**  **2*** | **ASV**  **132*** |
| GammaA^a^ | F | TTATGATGTTCTAGGTGATGTG | 0 | 4 | 4 | 5 | 4 |
|  | R | AACAATGTAGATTTCCTGAGCCTTATTC | 0 | 5 | 5 | 4 | 4 |
|  | P | TTGCAATGCCTATTCG | 0 | 2 | 2 | 2 | 2 |
| Gamma3 | F | AGAGCTTGATGACGTACTTA | 4 | 0 | 1 | 6 | 3 |
|  | R2 | G**A**AAGTTAATCGC**A**GTAATAAC |  | 0 | 1 |  |  |
|  | P | CTCTGGACCGCCTGCTTCTA | 5 | 1 | 0 | 0 | 0 |
| γETSP2^b^ | F | AGGCACGGTTGAGGATCTCG | 3 | 3 | 3 | 0 | 0 |
|  | R | CATACGCACCCTCTTCTTCAAGG | 4 | 2 | 4 | 0 | 0 |
|  | P | TTGGATGTGCCGGTCGCGGTGTTA | 3 | 1 | 1 | 0 | 2 |

^a^ Langlois et al. 2008 [36], ^b^ Turk-Kubo et al. 2014 [45]

**Supplementary Table 2**: Cross-hybridization of closely related ASVs detected with three qPCR assays covering the GammaA, Gamma3 and γETSP2 phylotypes. Note that the Gamma3 reverse primer was modified (Gamma3-R2 reverse primer; see Suppl. Table 1) to cover the mismatches detected among the prevalent Gamma3 ASV recovered in this study. C_t_ values show that the GammaA assay did not cross-hybridize with any of the ASVs from the γETSP2 and Gamma3 clades. C_t_ values indicate the threshold cycle for the detection of 1 million *nifH* gene copies. Asterisks indicate ASVs belonging to the ten most abundant ASVs in the dataset.

| **Clade** | ***nifH* ASV** | **Gamma3-F+R2 (C_t_)** | **ETSP2 (C_t_)** | **GammaA (C_t_)** |
| --- | --- | --- | --- | --- |
| GammaA | ASV019 | - | - | 17.9 |
| Gamma3 | ASV018* | 18.6 | 41.06 | - |
| Gamma3 | ASV021 | 30 | - | - |
| γETSP2 | ASV002* | - | 17.32 | - |
| H | ASV132* | - | 19.3 | - |
