# Supplementary material for: Diverse community of rhizobia-diatom symbioses fixes nitrogen in the South Pacific gyre
Source: ISME Commun. 2025 Nov 12;5(1):ycaf207. doi: 10.1093/ismeco/ycaf207 (PMC12667272; doi:10.1093/ismeco/ycaf207)
Supplement: Supplementary_Methods_ver2_ycaf207 [file supplementary_methods_ver2_ycaf207.docx]

**Supplementary Methods**

**to**

**Diverse community of** **Rhizobia-diatom symbioses fixes nitrogen in the South Pacific Gyre**

Mertcan Esti^1,‡^, Miriam Philippi^1,4,‡^, Julia Duerschlag^1,5^, Timothy G. Ferdelman^1^, Jennifer Tolman^2^, Julie LaRoche^2^, Clara Martínez-Pérez^1,6^, Gaute Lavik^1^, Bernhard Tschitschko^1,7^, Hon Lun Wong^1^, Alexandra Kraberg^3^, Sten Littmann^1^, Abiel T. Kidane^1^, Wiebke Mohr^1,*^, Marcel M. M. Kuypers^1^

***N_2_ fixation rate measurements***

N_2_ fixation rates were measured at six depths in the upper water column (surface waters to below the deep chl *a* maximum, DCM) at stations 2, 4, 6, 8, 10, 12, 14 and 15, in surface waters and at ~ 100 m depth at station 1, and in surface waters at stations 3, 5, 7, 9, 11 and 13. Rates were determined using the bubble removal technique [34, 35] simultaneously with primary production rates that are reported in Duerschlag et al. [31]. After collecting water from the CTD-rosette into the incubation bottles and the addition of the ^13^C-DIC tracer, the incubation bottles (~4.5-L) were closed headspace-free, and 10 mL of ^15^N_2_ gas were added to each of the triplicate bottles, which were then gently agitated for about 20 min. Afterwards, the gas bubble was removed, replaced with depth-specific seawater and the bottles were capped headspace-free. Bottles were incubated in on-deck incubators, cooled with a continuous flow of surface seawater. Light in the incubators was adjusted to three light levels with Lee filter foils (724 Ocean Blue for the upper two depths, 132 Special Medium Blue for the middle two depths, and 071 Tokyo Blue for the lower two depths; a detailed discussion on light levels can be found in the supplementary material of Duerschlag et al. [31]. At the end of the ~24-h incubation period, subsamples for enrichments of ^15^N in the N_2_ pool were taken from each individual incubation bottle, and enrichments were measured onboard using a membrane inlet mass spectrometer (MIMS; GAM200, IPI). Enrichment levels ranged from 3.56 to 10.57 at% ^15^N with a mean and standard deviation of 7.14 ± 1.14 at% ^15^N, respectively (with enrichment levels >5 at% in almost 90% of the incubations). Samples for elemental and isotopic analysis of the biomass (~3.7- 4.7 L) were collected onto pre-combusted (450 °C for 4-6 h) GF/F filters (Whatman) and stored at -20 °C until further analysis. Subsamples (100-200 mL) for fluorescence in situ hybridization and single-cell analyses were fixed with 1% (w/v) methanol-free paraformaldehyde for 2 h at room temperature or overnight at 4 °C (~12-24 h), filtered onto gold (Au)-sputtered (40 nm) polycarbonate filters (type GTTP; pore size: 0.2 µm; diameter: 25 mm; Millipore) and stored at -20 °C for later analysis.

GF/F filters were dried at 60 °C for ~ 1 h, acidified overnight over fuming hydrochloric acid (HCl; 37%), re-dried for ~ 2 h at 60 °C, and pelletized in tin cups. Particulate organic nitrogen concentration (PON) and its isotopic composition was determined using an elemental analyzer (Thermo Flash EA, 1112 Series) coupled to a continuous-flow isotope ratio mass spectrometer (Delta Plus XP IRMS; Thermo Finnigan, Dreieich, Germany) using the same filters and processing as described in Duerschlag et al. [31]. In detail, samples of high estimated biomass were split into half, and one half was analyzed. Samples with PON concentrations that were estimated to be too low to be precisely quantified were spiked with 10 µL of a caffeine standard (equivalent to 4.37 µg of N) prior to pelletizing into tin cups. Twenty-seven blank filters – also spiked with a 10 µL caffeine standard – were used to mass-balance PON and the atom% ^15^N of the samples treated with the caffeine spikes. Caffeine-spiked and half samples are indicated in the MQR data sheet (Suppl. Data File 1), and the values given in the table are after mass balancing. The caffeine-spiked blank filters were also used to establish the detection limits, i.e., three times the standard deviation (0.001373 at% ^15^N = 3 SD; ≈3.76 ‰ δ^15^N) of the mean at% ^15^N in the caffeine-spiked blank filters. The detection limit for each set of triplicate incubation bottles was set as the natural abundance value plus the three standard deviations. The natural abundance of ^15^N in the biomass was determined from single untreated controls that were incubated together with the triplicate ^15^N_2_ and ^13^C-DIC amended bottles and processed as above. N_2_ fixation rates were calculated from the incorporation of ^15^N_2_ into biomass according to Großkopf et al. [83]. Slightly negative rates were set to zero. Average rates were considered below the detection limit if the average rate was below the average detection limit of the triplicate set; averages below detection were set to zero for further analysis.

***Nucleic acid sampling and extraction***

Two liters of water were filtered onto polyvinylidene fluoride (PVDF) membrane filters (0.22 µm pore size, 47 mm diameter; Durapore), frozen immediately at -80 °C and stored at -80 °C. DNA was extracted using the Qiagen AllPrep DNA/RNA Mini Kit with the following modifications according to Langlois et al. [36]. The cryotube containing the filter was submerged in liquid nitrogen for about 30 s and the filter was then crushed with a sterile pipette tip. To facilitate lysis of cells, 200 µL of lysozyme (5 mg mL^-1^) were added to each cryotube. The tubes were vortexed and incubated for 10 min at room temperature. Kit lysis buffer (350 µL) was added, followed by an additional vortexing step. The lysate was transferred to a QiaShredder column (Qiagen). The samples were then further processed according to the manufacturers’ instructions. DNA was eluted in kit-supplied EB buffer and stored at -80 °C until further processing.

**nifH *amplicon sequencing and analysis***

Partial *nifH* genes were co-sequenced with partial 16S rRNA genes after PCR amplification, and sequences were separated based on primer sequences (16S rRNA primers V4-V5 versus *nifH* primers). The amplicon sequence variants (ASV) of the 16S rRNA gene sequences were reported in Duerschlag et al. [31] and can be found at the NCBI Sequence Read Archive under Bioproject number PRJNA670604.

Prior to *nifH* amplicon sequencing, the extracted DNA samples were screened for the presence of *nifH* genes as follows. Partial *nifH* genes were amplified from DNA by a nested PCR approach with the primer pairs nifH3/4 (PCR1) and nifH1/2 (PCR2) [84]. PCR1 reactions were run in a 25 µL volume composed of: 1x buffer (Qiagen), 4 mM MgCl_2_ (Qiagen), 120 µg mL^-1^ Bovine Serum Albumin (BSA; New England Biolabs), 0.025 U/µL HotStar *Taq* (Qiagen), 2.5 µL extracted DNA template, 800 nM each of dNTPs (Invitrogen) and nifH3/4 primers (Integrated DNA Technologies). PCR2 reactions were run in a 10 µL volume composed of 3 mM MgCl_2_ (Qiagen), 120 µg mL^-1^ BSA (New England Biolabs), 0.025 U/µL HotStar *Taq* (Qiagen), 1 µL of the PCR1 reaction as template, and 800 nM each of dNTPs (Invitrogen) and nifH1/2 primers (Integrated DNA Technologies). Molecular biology-grade water (Invitrogen) was used to make up the reaction volume, and in a no-template control. Thermocycler settings for PCR1 were: 95°C for 15 min, 35 cycles of [1 min each at 95°C, 45°C, and 72°C], then 72°C for 10 min. For PCR2, the annealing temperature and the number of cycles were set to 54°C and 28, respectively. Final PCR products of the expected size (359 bp) were confirmed via agarose gel electrophoresis, and only samples with visible PCR product were included for *nifH* amplicon library preparation and sequencing.

For the library preparation, a second PCR1 (PCR1d) was performed for *nifH*-positive samples using DNA diluted 1:10 to counteract amplification bias. Equal volumes of PCR1 and PCR1d were combined as template for a 25 µl PCR2 using *nifH* fusion primers (Integrated DNA Technologies) combining the nif1/2 primer sequences with Illumina adapters and barcodes [85]. Thermocycler settings were as above with an annealing temperature of 52 °C and 35 amplification cycles. The final barcoded products were cleaned and normalized with the Just-a-Plate 96 PCR Purification and Normalization kit (Charm Biotech). Samples were multiplexed at equal volumes and loaded into the Illumina MiSeq platform as a 20 pM final denatured library according to manufacturer’s instructions (see [86]). Tag sequencing of the *nifH* DNA amplicons was carried out using 2 x 300 bp paired-end v3 chemistry at the Integrated Microbiome Resource (IMR), Dalhousie University. The raw Illumina paired-end reads of *nifH* were processed with the QIIME pipeline [37] using the Microbiome Helper workflow of the IMR [38]. Briefly, paired-end reads were separated from co-sequenced 16S rRNA gene sequences using amplicon-specific primer sequences. Primers were trimmed using *cutadapt* [40], then reads were stitched together using PEAR [40] and denoised into ASVs using *deblur* [41] with a trim length of 325 bp and a *nifH* reference set [42]. Sequencing bleed-through was minimized by removing singletons and ASVs with a relative abundance of <0.1% mean sample depth [38].

***Phylogenetic analyses***

For *nifH* phylogenetic analyses, *nifH* reference sequences were collected. These included the single best hit for each *nifH* amplicon sequence variant (ASV) in a blastn search [43] against the NCBI Nucleotide collection (nt; accessed in April 2025), 20 *nifH* amplicon sequences from plankton-associated N_2_-fixers [44], 37 *nifH* sequences from heterotrophic bacterial diazotrophs (HBDs) [24], 26 *nifH* sequences representing different “Gamma” clusters [13, 22, 45], and the single best blastn hit for each *nifH* ASV against a custom full-length *nifH* blast database. This custom full-length *nifH* blast database was based on the full-length *nifH* sequences (over 885 bp) by Heller et al. [46] (last updated in 2022) with the addition of two *Ca*. Tectiglobus *nifH*, namely *Ca*. T. diatomicola (GCA_039583345) and *Ca*. T. profundi (GCA_039793665).

We constructed both nucleotide-based and amino acid-based phylogenetic trees. For the nucleotide-based tree, the *nifH* ASVs (excluding those that did not translate into an uninterrupted amino acid sequence, see below) and collected reference sequences were aligned with Mafft [87], and IQTree2 was used to construct a maximum-likelihood tree with 1000 bootstraps, utilizing ModelFinder to find the best-fit model [88, 89]. To construct a maximum-likelihood tree with NifH amino acid sequences, the collected *nifH* sequences were translated into amino acid sequences using seqkit [90]. Out of the 180 ASVs recovered, six were removed as no reading frame provided an uninterrupted amino acid sequence. The resulting 174 amino acid sequences (some of which were identical) and collected reference sequences were then aligned with Mafft [87]. Subsequently IQTree2 was used to construct a maximum-likelihood tree with 1000 bootstraps, using ModelFinder to find the best-fit model [88, 89]. The amino acid-based tree was used to provide a robust, conservative approach to broader phylogeny of recovered *nifH* amplicon sequences while the nucleotide-based tree was used to study clades within the group ‘Marine 1’ as amino acid-based phylogeny can disguise clades, as can be observed for UCYN-A clades [47]. Trees were visualized and pruned (where applicable) in iTol [48]. Assignment of ASVs to the canonical *nifH* clusters was done using the CART annotation tool [49]. The taxonomy of reference sequences is given only for those *nifH* sequences of known origin (using GTDB (Genome Taxonomy Database) taxonomy) while reference sequences of unknown taxonomic origin were designated as ‘unknown’.

Sequences that fell into the group ‘Marine 1’ as described by Langlois et al. [22], which also contains *Ca*. T. diatomicola and *Ca*. T. profundi, were further used for clustering analyses into clades. Starting at the common node, branches were sequentially analyzed for ASV sequence similarity, and clades were defined by 95% similarity with alignment coverage > 90% using CD-HIT [91]. This clustering resulted in 30 clades, many of which consisted of only 2 or 3 ASVs, and several ASVs that did not fall into a clade. For further description, we focused on those clades that contained either of the *Ca*. Tectiglobus species and/or any of the ten overall most abundant ASVs. Several of these clades contained reference sequences previously described as ‘gamma’ phylotypes [22]. For easier visualization of the clades within ‘Marine 1’, the *nifH* phylogenetic tree was pruned from the aforementioned best-fit-model tree using iTol [48].

The *nifH* sequence data can be found under Bioproject number PRJEB92104 at the European Nucleotide Archive (ENA) and ASVs can be found in Suppl. Data File 2. Tree files for both the nucleotide-based and amino acid-based trees can be found as Suppl. Data Files 3 and 4.

***Quantitative PCR***

Three previously published qPCR assays were used to confirm the presence/absence of ASVs belonging to the clades A/B, 3 and ETSP2. In addition, representative synthetic ASV sequences were used to assess the clade specificity of the qPCR assays. The ASV sequences selected as gBlock (double stranded synthetic DNA) standards corresponded to ASV2 and ASV132 for the ETSP2 and closely related H clade, respectively, ASV18 and ASV 21 for the ‘Marine 1-3’ clade, and ASV19 for the GammaA clade. The phylotype ‘ETSP2’ was quantified using the previously published quantitative PCR (qPCR) assay [45]. The ETSP2 assay reaction volume (18 μL total) was composed of: 9 μL of 2X TaqMan Environmental PCR master mix (Applied Biosystems), 0.36 μL of 10 μM PrimeTime 6-FAM/ZEN/3’IB probe (Integrated DNA Technologies), 0.72 μL each of forward and reverse 10 μM primers (Integrated DNA Technologies), 0.18 μL of BSA (20 mg mL^-1^; New England Biosystems), 2.02 μL of molecular biology-grade water (Invitrogen) and 5 μL of 1:2 diluted DNA template. Cycling conditions were as follows: 50 °C - 2 min, 95 °C - 10 min and then 45 cycles of 95 °C - 15 s and 60 °C - 1 min. A published Gamma3 qPCR assay was modified slightly to detect and quantify members of the ‘Marine 1-3’ clade [13] found in our study. Within the clade ‘Marine 1-3’, the most abundant ASV (ASV18) and the ASV most closely related to the Gamma3 reference sequence (ASV21) have mismatches to the primers and/or probe of the existing qPCR assay. We therefore modified the reverse primer (Gamma3-R2: 5’-GAAAGTTAATCGCAGTAATAAC-3’) to better target our ASVs resulting in one mismatch in the probe for ASV18 and one mismatch in each primer for ASV21 (Suppl. Table 1). The revised assay was used in an 18 µL reaction as above, but with 1.44 μL each of forward and reverse 10 μM primers (Integrated DNA Technologies) and 0.58 μL of molecular biology-grade H_2_O (Invitrogen). Cycling conditions were as above. The GammaA assay was carried out as published [36]. Cycling conditions were as above. All analyses were run on a StepOnePlus real-time PCR thermocycler (Applied Biosystems) with the accompanying StepOne software and standard settings used to determine gene copies per reaction. Average individual reaction efficiencies of the ETSP2, Gamma3 and GammaA were all greater than 95%. DNA used for standard curves (serially diluted and run in duplicate) and cross-reactivity tests (run in duplicate) were ordered as individual gBlocks gene fragments (Integrated DNA Technologies) and corresponded to ASVs 2, 18, 19, 21, 132, as listed above. These DNA fragments were quantified shortly before each use via a Qubit 4 fluorometer (Invitrogen) with a Qubit 1× dsDNA High-Sensitivity assay (Invitrogen). The limit of quantification for qPCR assays was 120-180 copies L^−1^. C_t_ values from cross-hybridization tests with the various gBlocks (Suppl. Table 2) show that the qPCR assays were specific to the targeted clades. In particular, no cross-clade interferences were detected for the γETSP2 and other Gamma assays. A full table of mismatches between all ASVs in the group ‘Marine 1’ and primers/probes can be found in Suppl. Data File 5.

***Recovery of the full-length 16S rRNA gene sequence of* Ca. *Tectiglobus profundi***

The metagenome-assembled genome (MAG) of the closest relative of *Ca*. T. diatomicola, i.e., *Ca*. T. profundi (GCA_039793665) only contained a partial (1108 bp) 16S rRNA gene sequence [26]. In order to assess the specificity of all probes used during fluorescence in situ hybridization (see below), we iteratively extended its genome with the aim of recovering the full-length 16S rRNA gene sequence. All sixty-three short read metagenomes from the North Pacific study [92, 93], which reported the original MAG (GCA_013214245) lacking *nif* genes, were downloaded and trimmed using bbduk (ktrim=l trimq=15 qtrim=rl minlength=50 mink=11) [94]. The trimmed reads were then mapped to GCA_039793665 with a 95% identity cutoff. The five samples (SRR7648289, SRR7648288, SRR7648332, SRR7648273 and SRR7648294) in which GCA_039793665 had the highest abundance were used to iteratively extend and refine the genome using a custom script described in Tschitschko et al. [26]. After 11 iterations, the 16S rRNA sequence was extended from 1108 bp to 1452 bp, the number of contigs were reduced from 25 to 8, and maximum contig length was increased from 234,532 bp to 839,261 bp, overall indicating increased gene contiguity. CheckM2 [95] was used to estimate genome completeness and contamination, and completeness increased from 99.96% to 100% while contamination remained at 0%. The refined genome of *Ca*. T. profundi retains its small genome size (1.85 Mb) and its reduced GC content (39%), and had an average nucleotide identity (ANI) of 99.92% to the original version of the *Ca*. T. profundi genome. The refined, full-length 16S rRNA gene sequence can be found in Suppl. Data File 6.

***Fluorescence in situ hybridization and microscopy***

Subsamples from the end of the incubation experiments as well as plankton net samples were used to visualize *Ca*. Tectiglobus spp. via fluorescence in situ hybridization (FISH) in a double CARD-FISH approach according to Tschitschko et al. [26] and adhering to standard procedures [50] with the following conditions. Endogenous peroxidases were inactivated with 3% H_2_O_2_ for 10 min at room temperature, followed by cell permeabilization using lysozyme (10 mg mL^-1^ in 0.05 M EDTA 0.1 M Tris-HCl, 37 °C for 30 min). In the first hybridization, horseradish peroxidase (HRP)-labeled probes Hypho825 and Hypho638 were used simultaneously along with their corresponding helper (Helper854) and competitor (Comp638) probes to increase accessibility and specificity, respectively. Hybridization was carried out at 46 °C for 1.5 h in hybridization buffer containing 45% formamide (FA), followed by signal amplification using tyramides (1 ng µL^-1^) labeled with Oregon Green 488 (OG488) at 46 °C for 20 min. After the first amplification, HRP probes were inactivated using 0.01 M HCl at room temperature for 10 min. In the second hybridization, probe Hypho 1147 and helper probe Helper1116 were hybridized at 46 °C for 1.5 h with a formamide concentration of 40%. Subsequent amplification was performed using tyramides labeled with Alexa594 (A594) under the same conditions (1 ng µL^-1^, 46 °C for 20 min). Filters were washed after each hybridization and amplification step for 10 min in wash buffer (20 mM Tris/HCl, 5 mM EDTA, 30 mM (first hybridization) or 46 mM (second hybridization) sodium chloride, 0.01% sodium dodecyl sulfate) and 1x phosphate-buffered saline (PBS). Finally, cells were counterstained with 4',6-diamidino-2-phenylindole (DAPI; 1 ng µL^-1^, 10 min at 4 °C in the dark). Filter pieces were mounted in a mixture of Citifluor and Vectashield (4:1 v/v) for microscopy.

Diatoms were considered to contain symbionts (FISH-positive cells) when fluorescence signals were substantially higher than background fluorescence within the same field and clearly distinguishable from negative controls hybridized with the NON338 probe (see [26]). Tyramide deposition did not always stain the entire cell as signal localization will depend on the distribution of ribosomes in a cell [96]. FISH signals were usually co-localized with signals of the nucleic acid stain DAPI. Diatoms containing FISH-positive cells were marked using a laser microdissection microscope (LMD 7000, Leica) and imaged using a Zeiss Axio Imager.M2 wide-field epifluorescence microscope equipped with a Zeiss Axiocam 506 mono camera and an xyz-stage. Scanning electron microscopy (FEI Quanta 250 FEG ESEM; Thermo Fisher Scientific) was used to subsequently determine host morphology.

From incubation samples, diatoms with FISH-positive cells were counted from 1-2 filter pieces of ~ 5 mm diameter each (cut from the 25 mm diameter filters), which equated to 19-31 mL of incubated seawater. A few of these diatoms were further analyzed for their single-cell activity using nanoscale secondary ion mass spectrometry (nanoSIMS; see below). For the plankton net samples, diatoms were only visualized but not quantified due to the uneven distribution of collected plankton on the filter material and the mesh size of the plankton net, likely not capturing all diatoms.

***Single-cell activities using nanoSIMS***

Nanoscale secondary ion mass spectrometry (nanoSIMS) analysis was performed using a NanoSIMS 50L (CAMECA, Paris, France) as previously described [26]. Marked filter areas with diatoms were briefly pre-sputtered with a strong defocused Cs^+^ primary ion beam to remove surface contamination and the outer silica wall of the diatom. The sample area was then rastered with a 1.5 pA Cs^+^ primary ion beam (beam diameter around 100 nm) at a raster size between 50 x 50 µm to 35 x 35 µm with a dwell time of 1 ms and with 256 pixel x 256 pixel image resolution. Negative secondary ions – ^12^C^-^, ^13^C^-^, ^19^F^-^, ^12^C^14^N^-^, ^12^C^15^N^-^, ^31^P^-^ and ^32^S^-^ – were collected simultaneously in electron multiplier detectors of the multi-collection system in up to 60 planes for each measurement. Acquired data was processed with Look@nanosims v2023 [57], including dead-time and drift correction before accumulating the recorded planes. On accumulated planes, regions of interest (ROIs) were defined by overlaying epifluorescence images of the diatoms with the secondary ion images of the nanoSIMS measurement. Isotopic ratios of ^13^C/^12^C and ^12^C^15^N/^12^C^14^N of the ROIs were determined, and only cells with a Poisson error of less than 5% were used for further analyses of single-cell rates. As possible isotopic dilution of ^15^N/^14^N and ^13^C/^12^C ratios due to CARD-FISH was not taken into account, the ratio values and resulting single-cell rates are considered conservative [97-99]. Single-cell rates were calculated based on the isotopic enrichment and cell biomass. Cell biomass was estimated from cell size (obtained from nanoSIMS and SEM imaging) which was converted to carbon mass using the prolate spheroid (symbionts) and prism-on-elliptic base (host) models [100] and the conversion factor by Khachikyan et al. [101] and Verity et al. [102], respectively. Nitrogen mass was calculated from carbon mass using the Redfield ratio of 6.6 C : 1 N.

**Supplementary References**

83. Großkopf T, Mohr W, Baustian T, Schunck H, Gill D, Kuypers MMM et al. Doubling of marine dinitrogen-fixation rates based on direct measurements. *Nature* 2012;**488**:361-364. https://doi.org/10.1038/nature11338

84. Zehr JP, Turner PJ. Nitrogen fixation: Nitrogenase genes and gene expression. *Methods In Microbiology* 2001;**30**:271-286. https://doi.org/10.1016/S0580-9517(01)30049-1

85. Ratten J-M. The diversity, distribution and potential metabolism of non-cyanobacterial diazotrophs in the North Atlantic ocean. Halifax: Dalhousie University, 2017

86. Fonseca-Batista D, Li X, Riou V, Michotey V, Deman F, Fripiat F et al. Evidence of high N_2_ fixation rates in the temperate northeast Atlantic. *Biogeosciences* 2019;**16**:999-1017. https://doi.org/10.5194/bg-16-999-2019

87. Katoh K, Misawa K, Kuma Ki, Miyata T. MAFFT: a novel method for rapid multiple sequence alignment based on fast Fourier transform. *Nucleic Acids Research* 2002;**30**:3059-3066.

88. Kalyaanamoorthy S, Minh BQ, Wong TK, Von Haeseler A, Jermiin LS. ModelFinder: fast model selection for accurate phylogenetic estimates. *Nature Methods* 2017;**14**:587-589.

89. Minh BQ, Schmidt HA, Chernomor O, Schrempf D, Woodhams MD, Von Haeseler A et al. IQ-TREE 2: new models and efficient methods for phylogenetic inference in the genomic era. *Molecular Biology and Evolution* 2020;**37**:1530-1534.

90. Shen W, Le S, Li Y, Hu F. SeqKit: a cross-platform and ultrafast toolkit for FASTA/Q file manipulation. *PLOS One* 2016;**11**:e0163962.

91. Fu LM, Niu BF, Zhu ZW, Wu ST, Li WZ. CD-HIT: accelerated for clustering the next-generation sequencing data. *Bioinformatics* 2012;**28**:3150-3152. https://doi.org/10.1093/bioinformatics/bts565

92. Boeuf D, Edwards BR, Eppley JM, Hu SK, Poff KE, Romano AE et al. Biological composition and microbial dynamics of sinking particulate organic matter at abyssal depths in the oligotrophic open ocean. *Proceedings of the National Academy of Sciences* 2019;**116**:11824-11832.

93. Poff KE, Leu AO, Eppley JM, Karl DM, DeLong EF. Microbial dynamics of elevated carbon flux in the open ocean’s abyss. *Proceedings of the National Academy of Sciences* 2021;**118**:e2018269118.

94. Bushnell B. BBDuk: Adapter. *Quality Trimming and Filtering https://sourceforge.net/projects/bbmap/* 2014

95. Chklovski A, Parks DH, Woodcroft BJ, Tyson GW. CheckM2: a rapid, scalable and accurate tool for assessing microbial genome quality using machine learning. *Nature Methods* 2023;**20**:1203-1212.

96. Hill JD, Papoutsakis ET. Species-specific ribosomal RNA-FISH identifies interspecies cellular-material exchange, active-cell population dynamics and cellular localization of translation machinery in clostridial cultures and co-cultures. *mSystems* 2024;**9**:e00572-00524.

97. Woebken D, Burow LC, Behnam F, Mayali X, Schintlmeister A, Fleming ED et al. Revisiting N_2_ fixation in Guerrero Negro intertidal microbial mats with a functional single-cell approach. *ISME Journal* 2015;**9**:485-496.

98. Musat N, Stryhanyuk H, Bombach P, Adrian L, Audinot J-N, Richnow HH. The effect of FISH and CARD-FISH on the isotopic composition of ^13^C-and ^15^N-labeled *Pseudomonas putida* cells measured by nanoSIMS. *Systematic and Applied Microbiology* 2014;**37**:267-276.

99. Meyer NR, Fortney JL, Dekas AE. NanoSIMS sample preparation decreases isotope enrichment: magnitude, variability and implications for single‐cell rates of microbial activity. *Environmental Microbiology* 2021;**23**:81-98.

100. Sun J, Liu D. Geometric models for calculating cell biovolume and surface area for phytoplankton. *Journal of Plankton Research* 2003;**25**:1331-1346.

101. Khachikyan A, Milucka J, Littmann S, Ahmerkamp S, Meador T, Könneke M et al. Direct cell mass measurements expand the role of small microorganisms in nature. *Applied and Environmental Microbiology* 2019;**85**:e00493-00419.

102. Verity PG, Robertson CY, Tronzo CR, Andrews MG, Nelson JR, Sieracki ME. Relationships between cell volume and the carbon and nitrogen content of marine photosynthetic nanoplankton. *Limnology and Oceanography* 1992;**37**:1434-1446.
